# Supplementary material for: Multi-Task Deep Learning for Surface Metrology
Source: Sensors (Basel). 2025 Dec 8;25(24):7471. doi: 10.3390/s25247471 (PMC12736692; doi:10.3390/s25247471)
Supplement: Supplementary file 1 [file sensors-25-07471-s001.zip › sensors-3996789-supplementary.pdf]

sensors

Article

Multi-Task Deep Learning for Surface Metrology

D. Kucharski [1,*], A. Gąska [2], T. Kowaluk [3], K. Stępień [4], M. Rępalska [3], B. Gapiński [1], M. Wieczorowski [1], M. Nawotka [5], P. Sobecki [6,5], P. Sosinowski [5], J. Tomasik [3] and A. Wójtowicz [5]

1 Poznan University of Technology, Poland
2 Cracow University of Technology, Poland
3 Warsaw University of Technology, Poland
4 Warsaw University of Technology, Poland
5 Central Office of Measures, Warsaw, Poland
6 National Information Processing Institute, Warsaw, Poland
* Correspondence: mailto:dawid.kucharski@put.poznan.pldawid.kucharski@put.poznan.pl

Supplementary Material

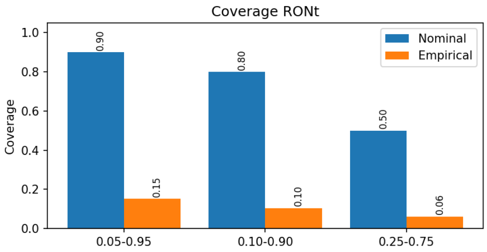

Figure S1: coverage plot ()

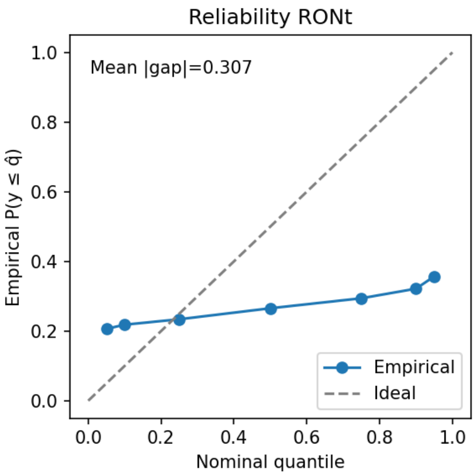

Figure S2: reliability plot ()

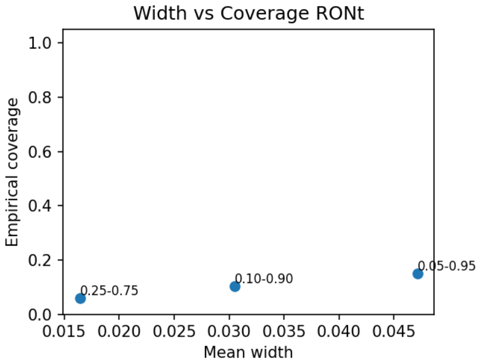

Figure S3: width vs coverage ()

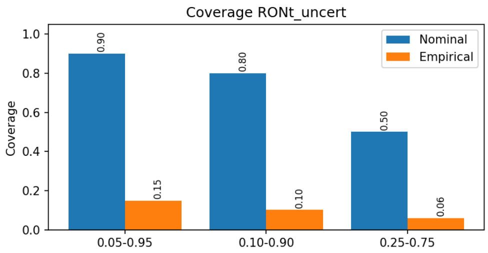

Figure S4: coverage plot ()

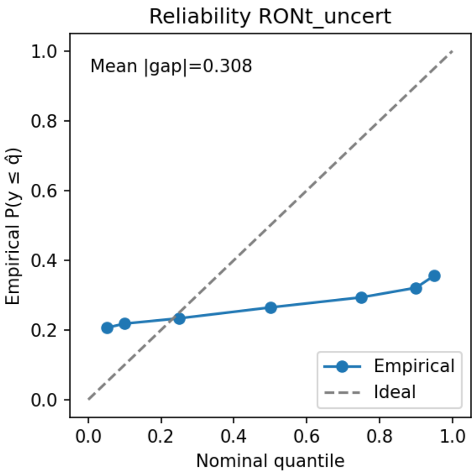

Figure S5: reliability plot ()

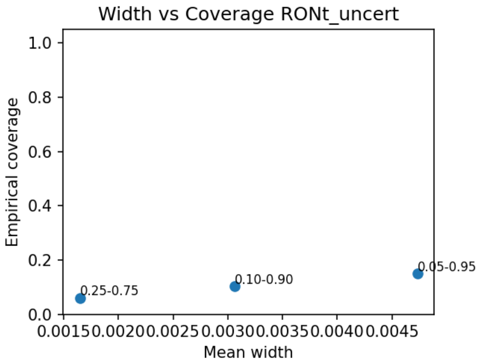

Figure S6: width vs coverage ()

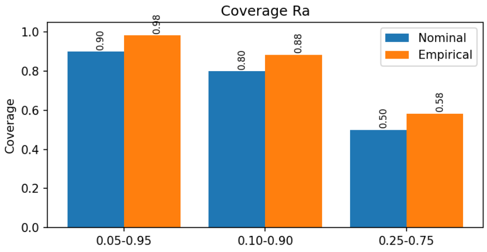

Figure S7: coverage plot ()

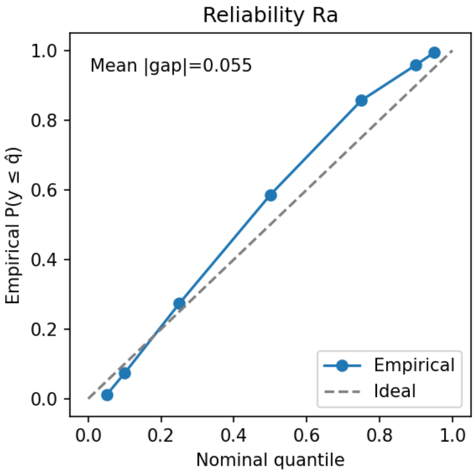

Figure S8: reliability plot ()

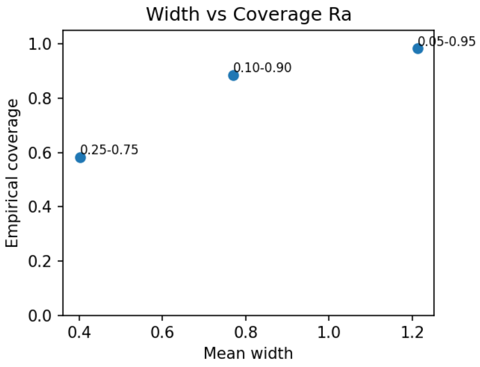

Figure S9: width vs coverage ()

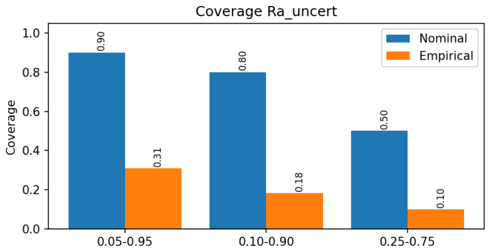

Figure S10: coverage plot ()

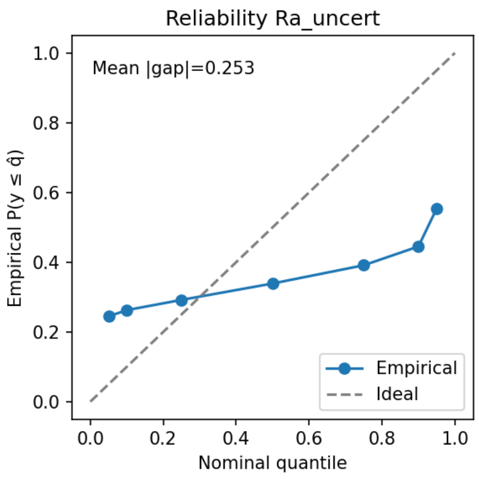

Figure S11: reliability plot ()

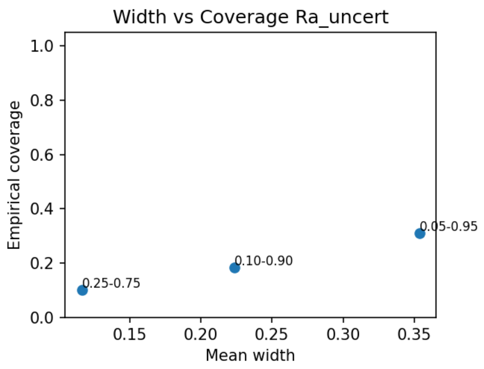

Figure S12: width vs coverage ()

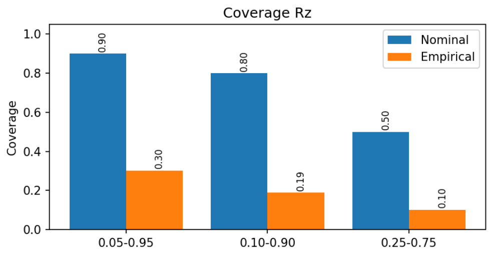

Figure S13: coverage plot ()

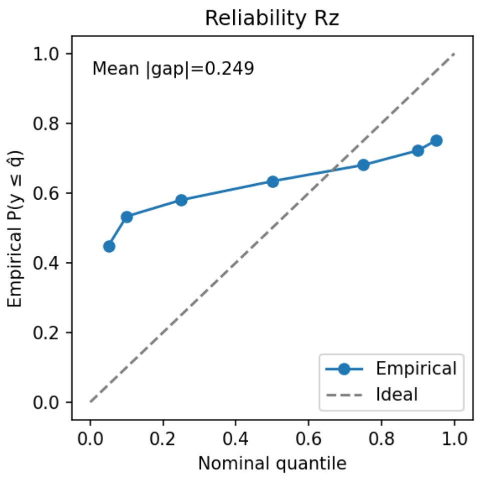

Figure S14: reliability plot ()

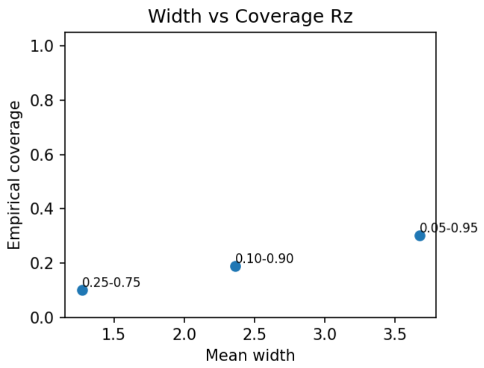

Figure S15: width vs coverage ()

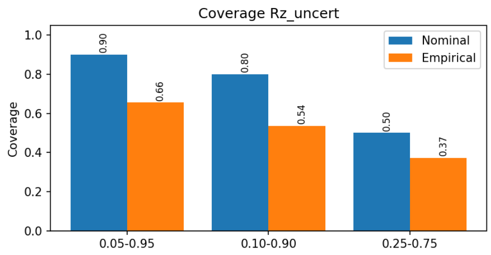

Figure S16: coverage plot ()

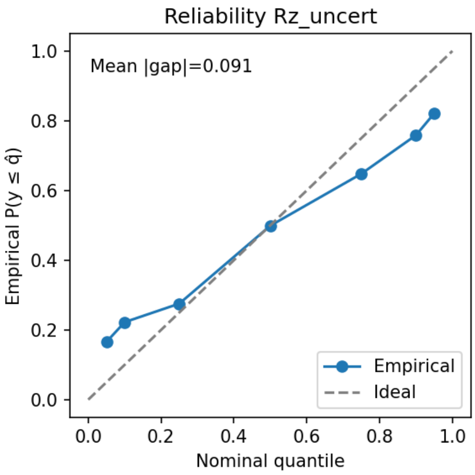

Figure S17: reliability plot ()

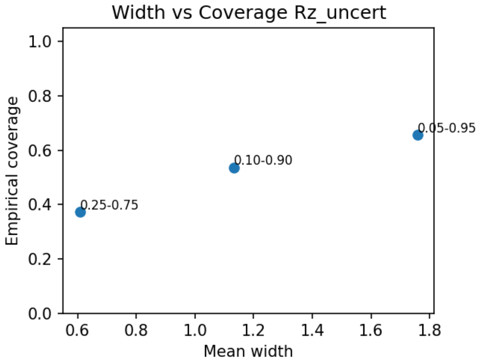

Figure S18: width vs coverage ()

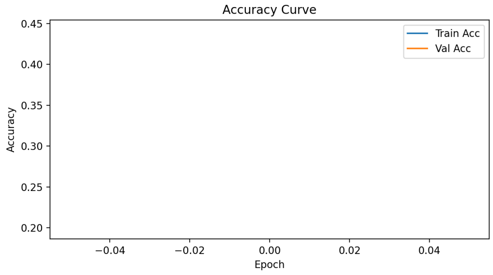

Figure S19: accuracy curve ()

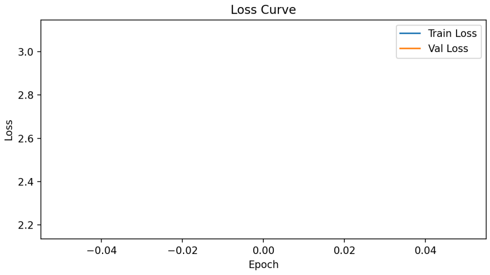

Figure S20: loss curve ()

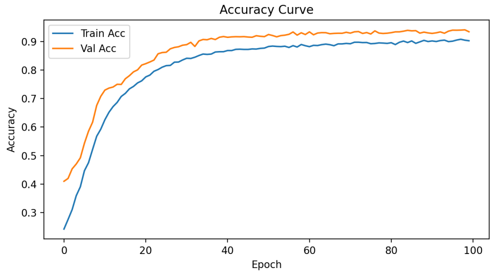

Figure S21: accuracy curve ()

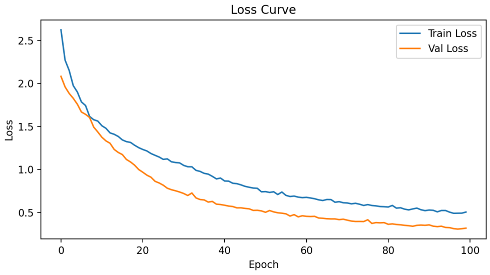

Figure S22: loss curve ()

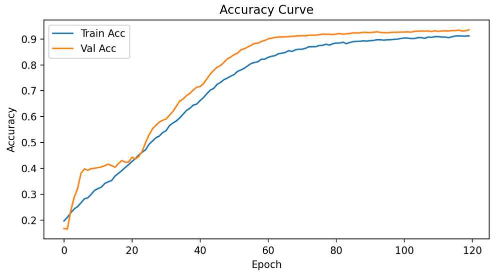

Figure S23: accuracy curve ()

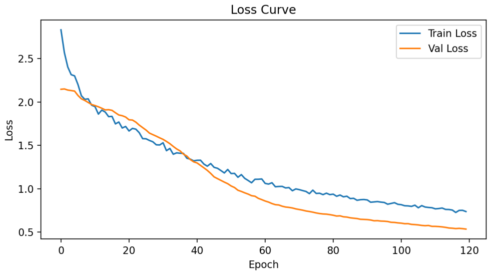

Figure S24: loss curve ()

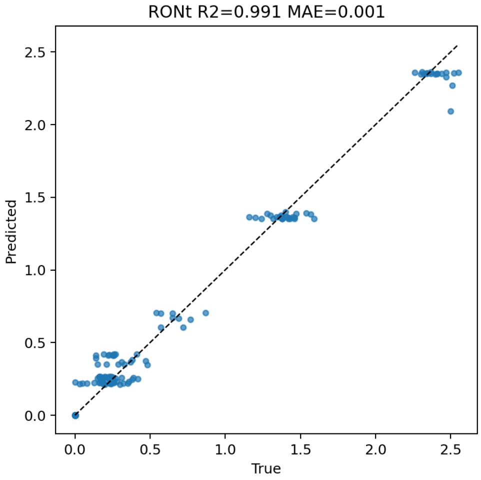

Figure S25: pred vs true ()

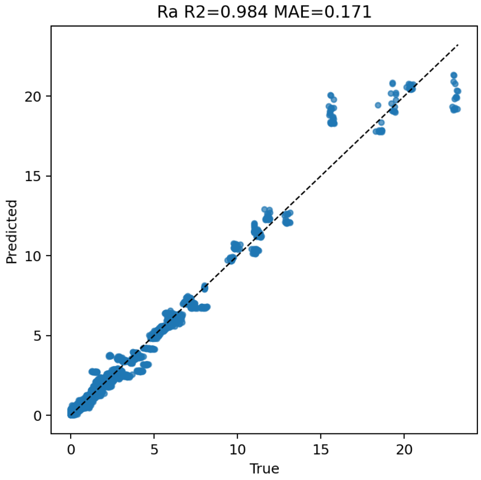

Figure S26: pred vs true ()

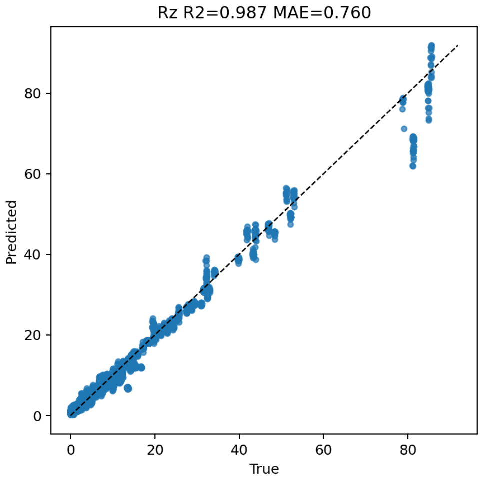

Figure S27: pred vs true ()

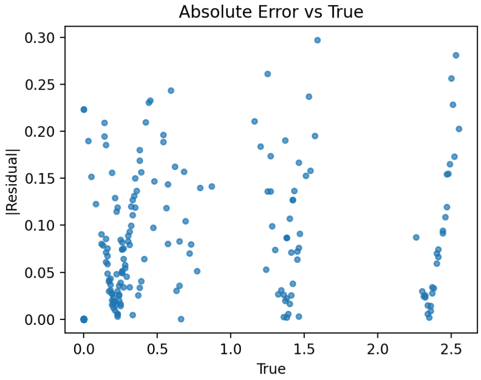

Figure S28: abs error vs true ()

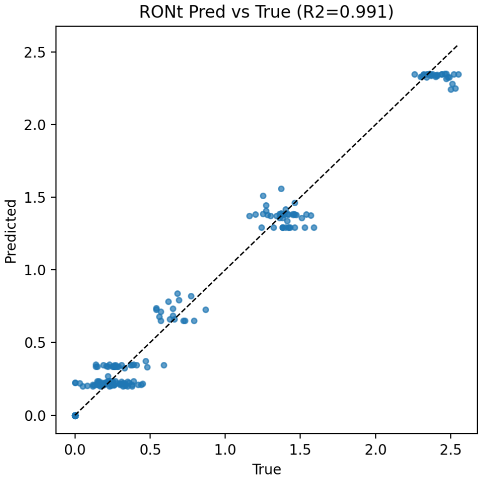

Figure S29: pred vs true ()

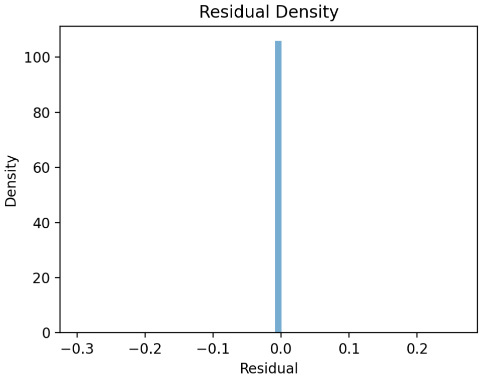

Figure S30: residual density ()

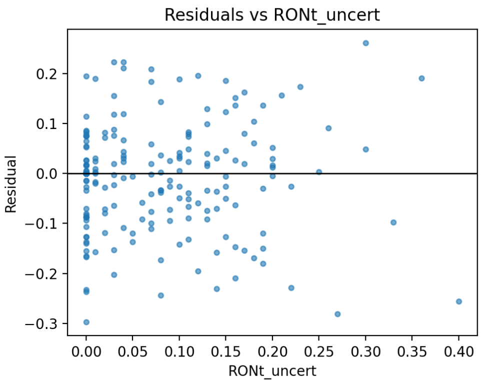

Figure S31: residuals vs top feature RONt uncert ()

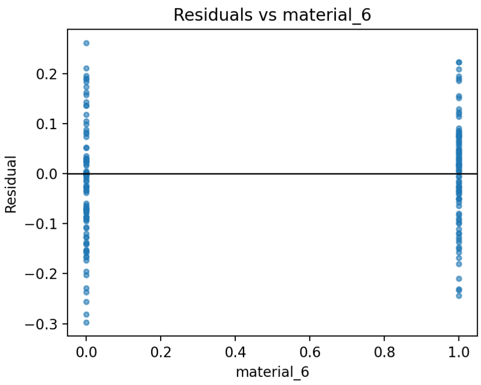

Figure S32: residuals vs top feature material 6 ()

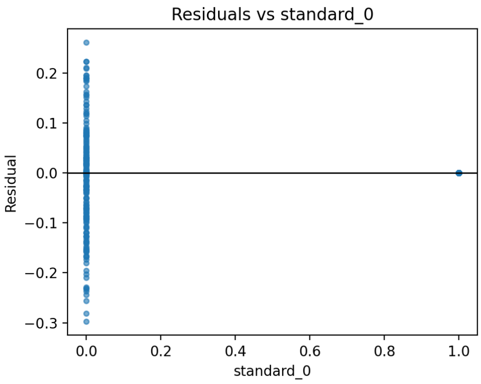

Figure S33: residuals vs top feature standard 0 ()

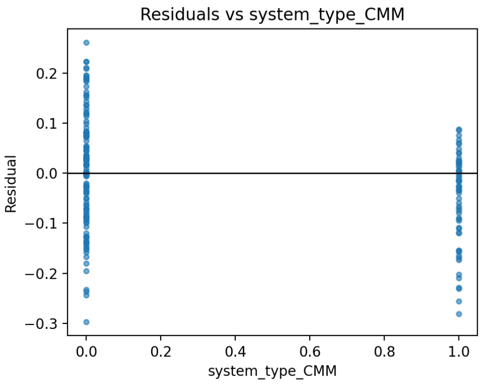

Figure S34: residuals vs top feature system type CMM ()

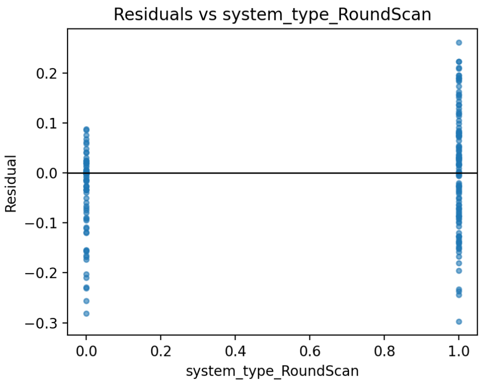

Figure S35: residuals vs top feature system type RoundScan ()

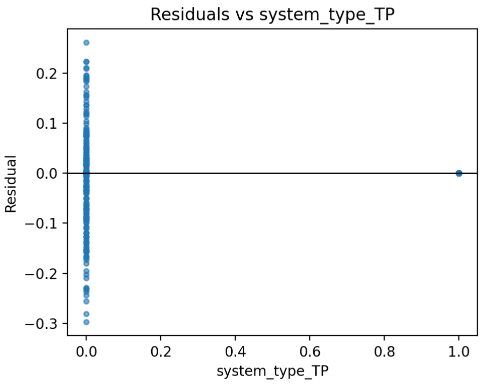

Figure S36: residuals vs top feature system type TP ()

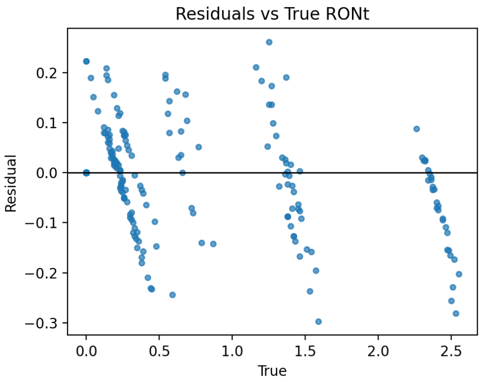

Figure S37: residuals vs true ()

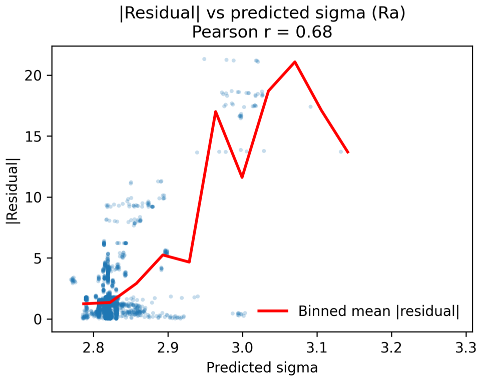

Figure S38: abs residual vs sigma Ra ()

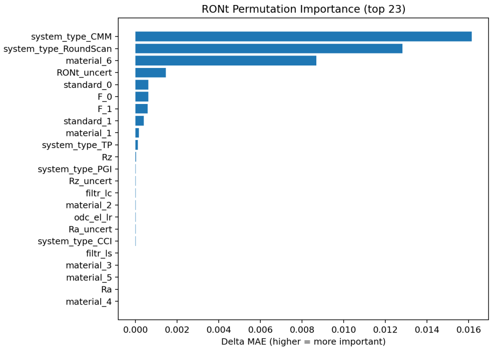

Figure S39: permutation importance ()

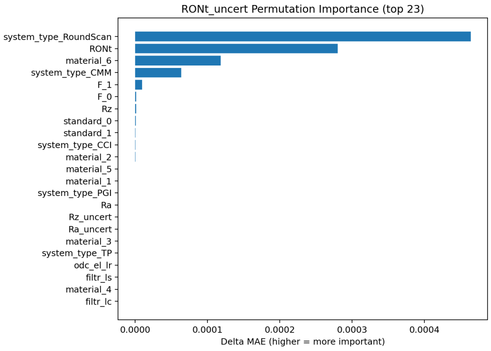

Figure S40: permutation importance ()

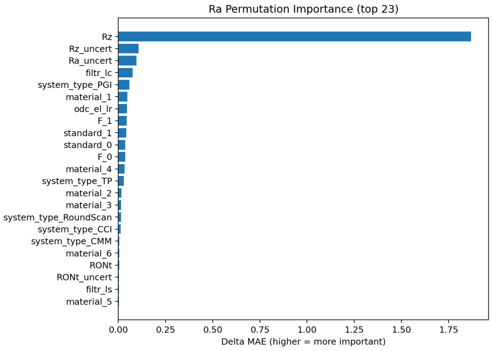

Figure S41: permutation importance ()

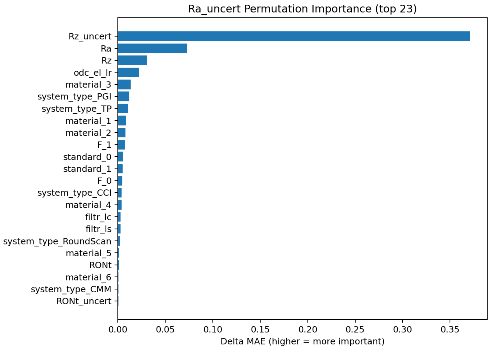

Figure S42: permutation importance ()

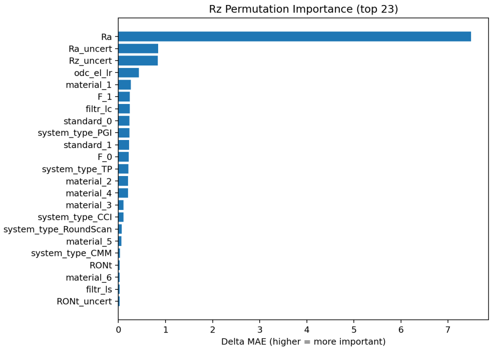

Figure S43: permutation importance ()

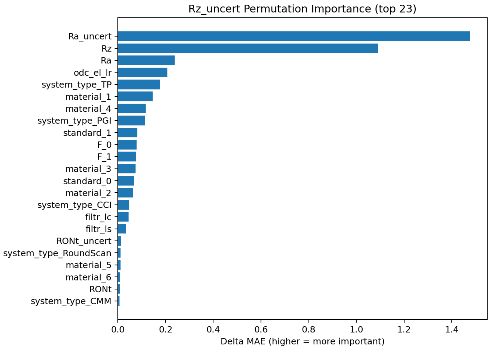

Figure S44: permutation importance ()

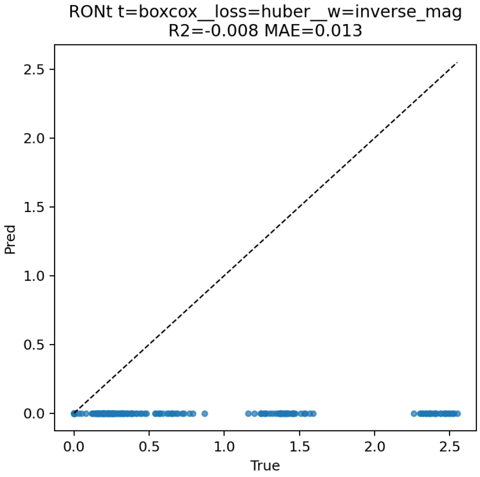

Figure S45: pred vs true ()

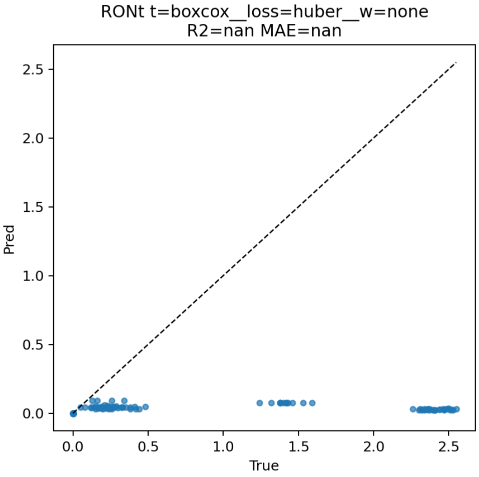

Figure S46: pred vs true ()

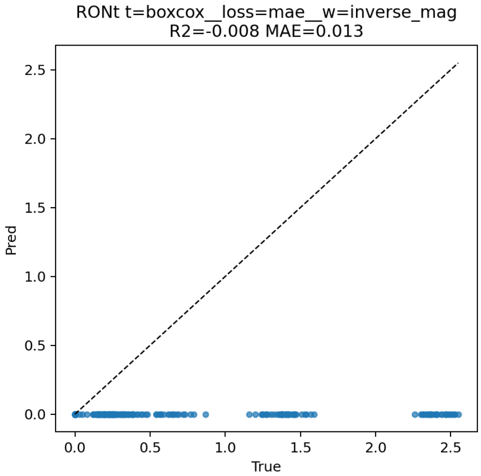

Figure S47: pred vs true ()

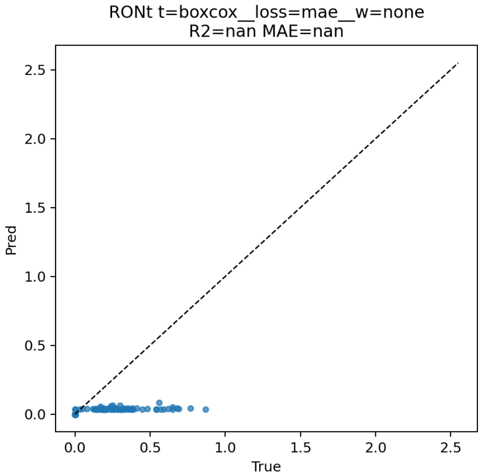

Figure S48: pred vs true ()

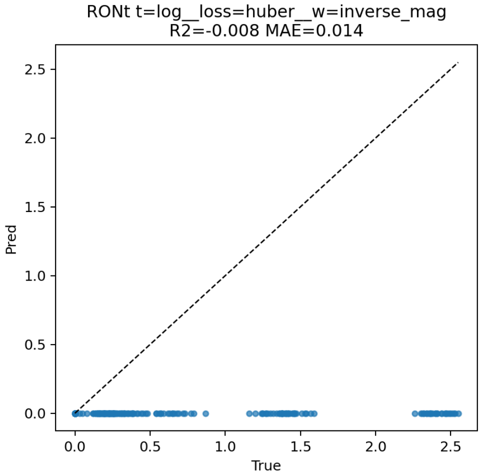

Figure S49: pred vs true ()

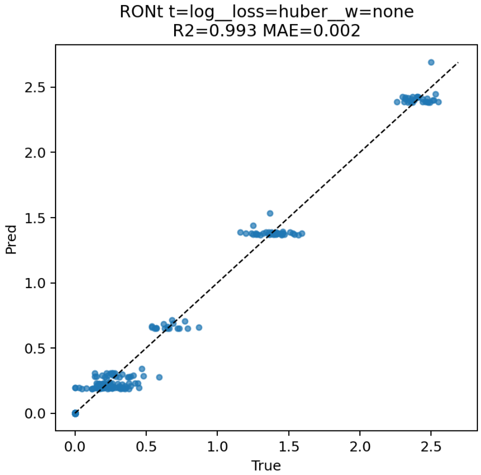

Figure S50: pred vs true ()

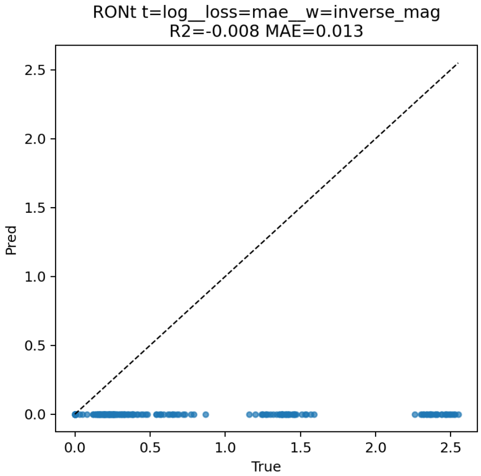

Figure S51: pred vs true ()

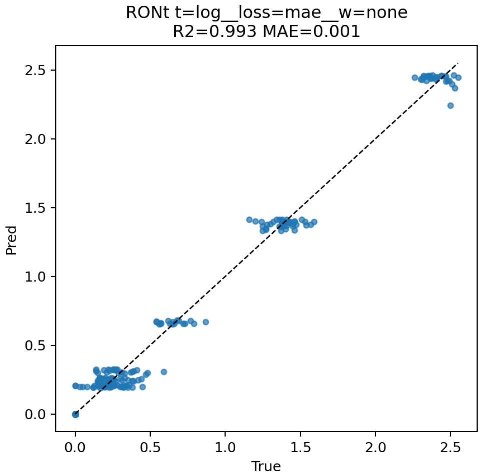

Figure S52: pred vs true ()

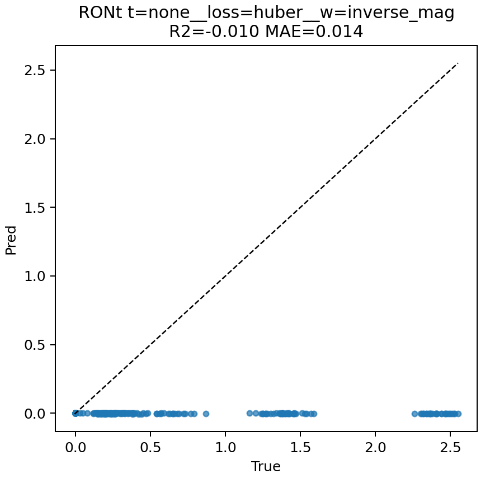

Figure S53: pred vs true ()

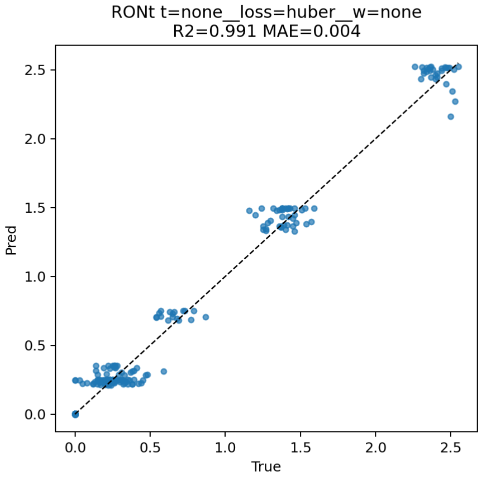

Figure S54: pred vs true ()

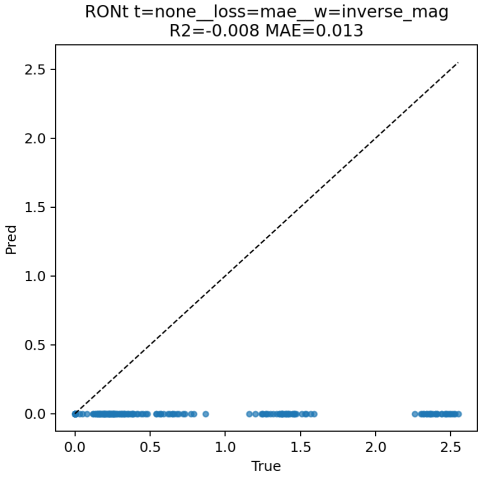

Figure S55: pred vs true ()

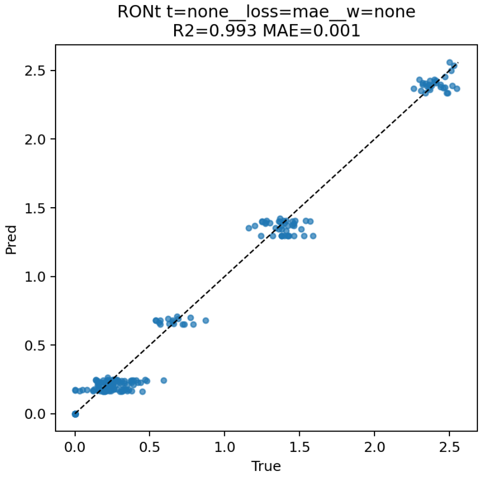

Figure S56: pred vs true ()

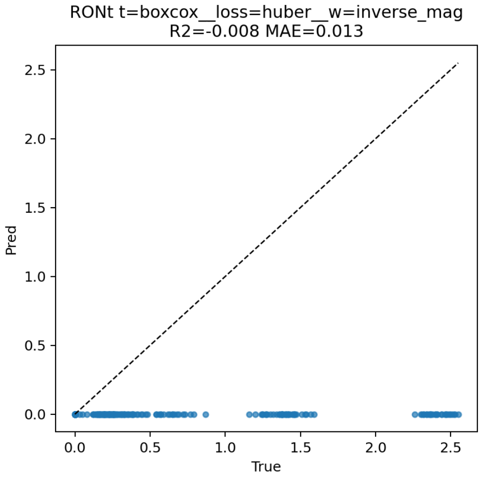

Figure S57: pred vs true ()

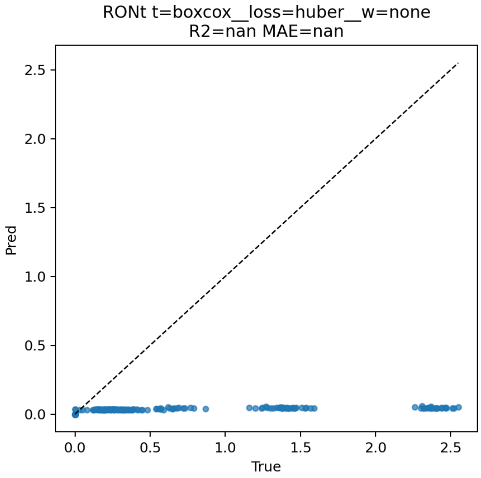

Figure S58: pred vs true ()

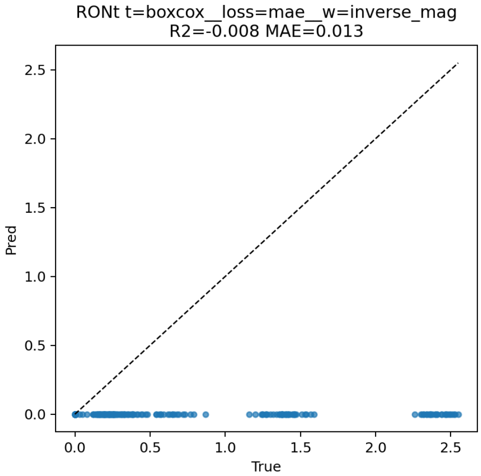

Figure S59: pred vs true ()

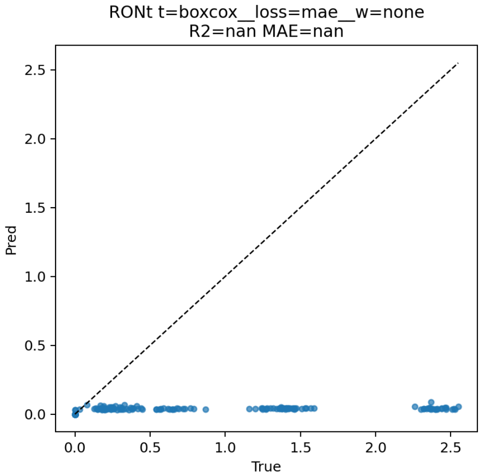

Figure S60: pred vs true ()

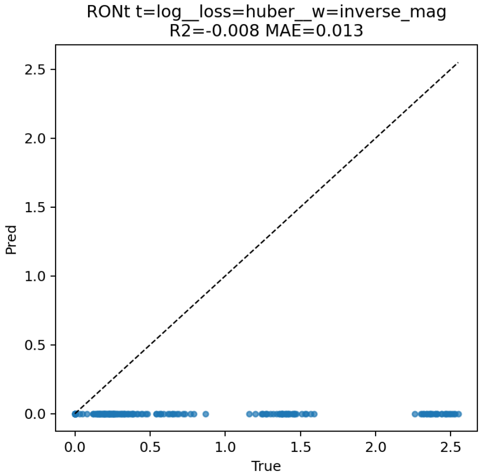

Figure S61: pred vs true ()

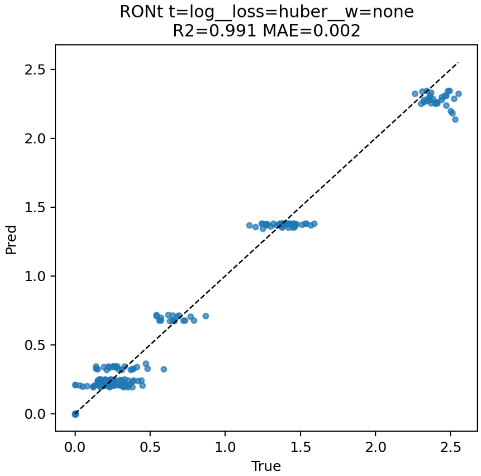

Figure S62: pred vs true ()

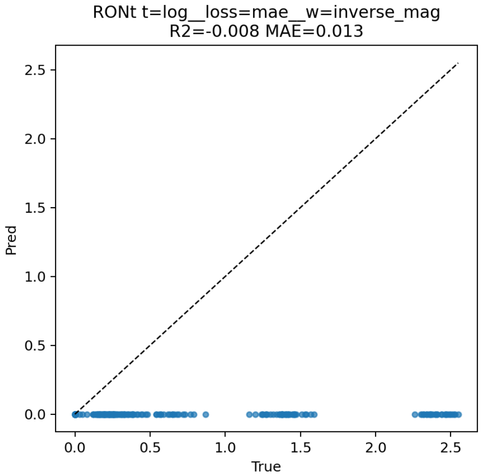

Figure S63: pred vs true ()

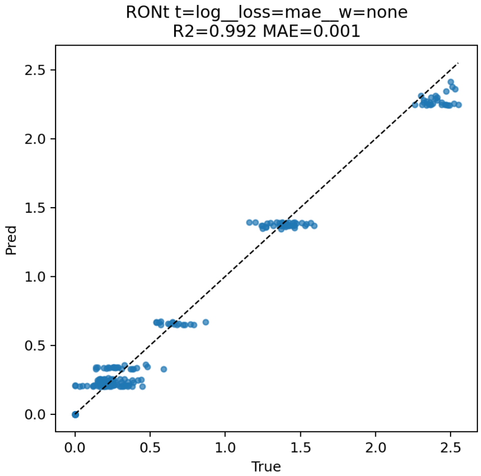

Figure S64: pred vs true ()

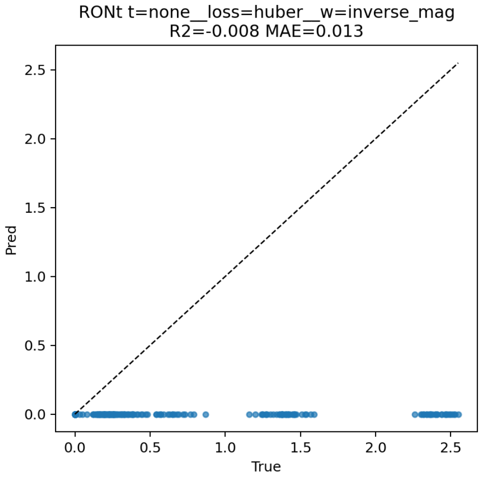

Figure S65: pred vs true ()

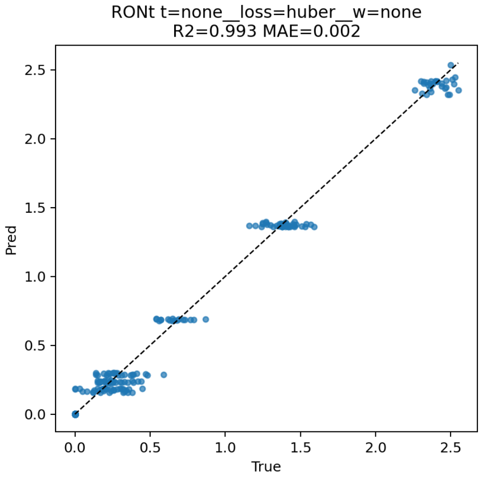

Figure S66: pred vs true ()

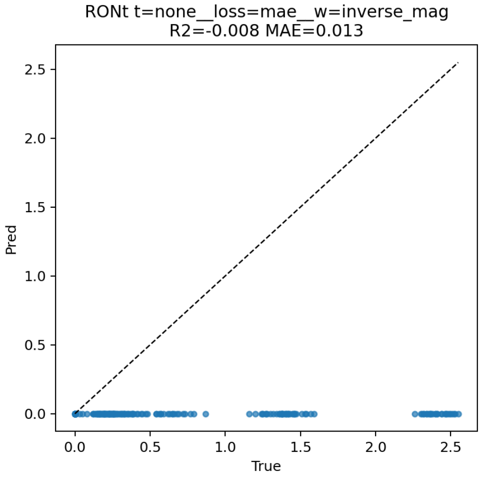

Figure S67: pred vs true ()

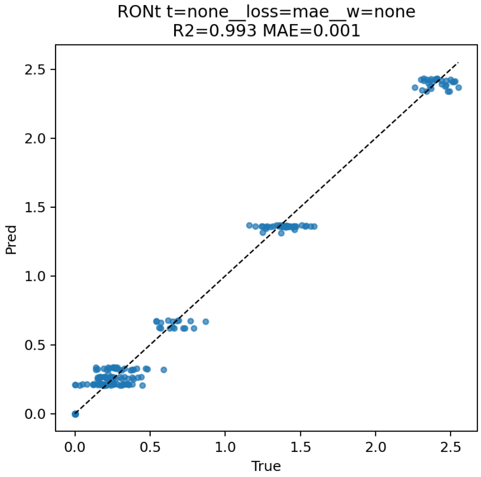

Figure S68: pred vs true ()

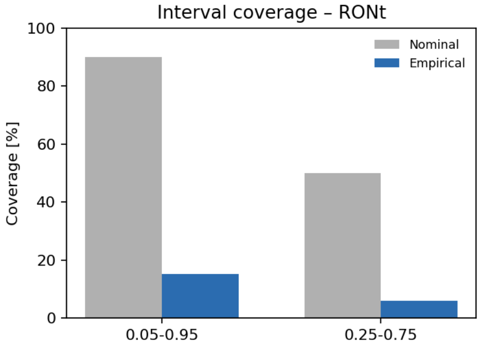

Figure S69: coverage RONt ()

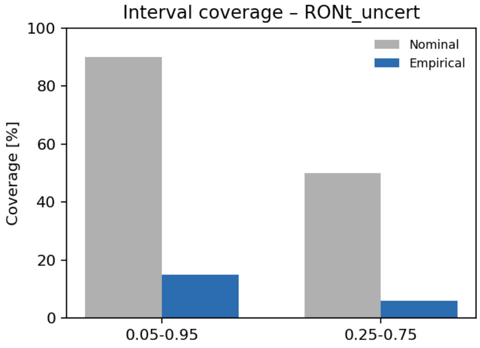

Figure S70: coverage RONt uncert ()

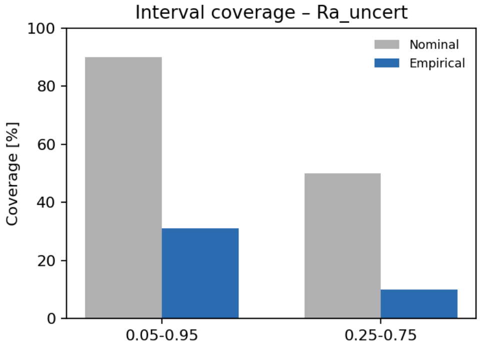

Figure S71: coverage Ra uncert ()

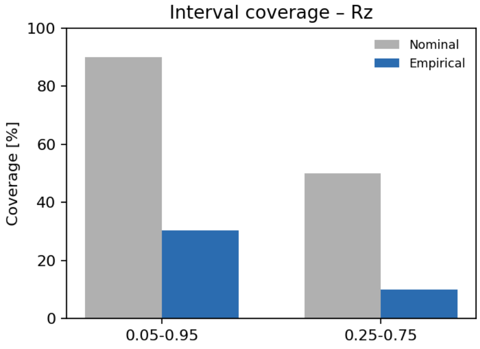

Figure S72: coverage Rz ()

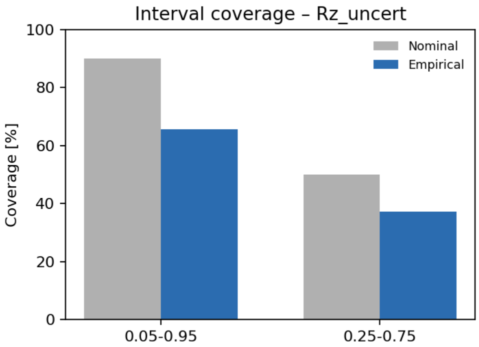

Figure S73: coverage Rz uncert ()

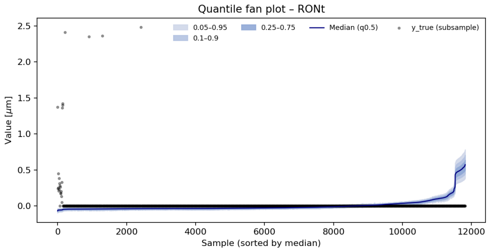

Figure S74: fan RONt ()

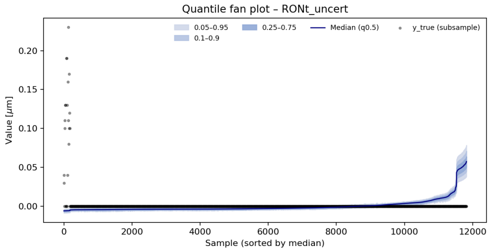

Figure S75: fan RONt uncert ()

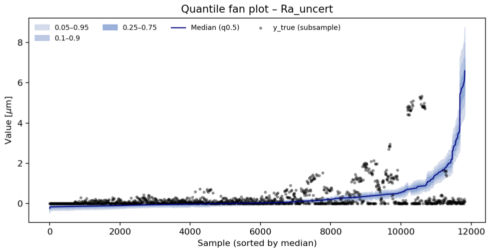

Figure S76: fan Ra uncert ()

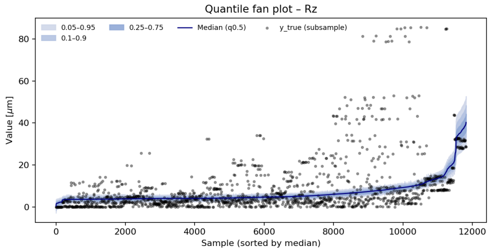

Figure S77: fan Rz ()

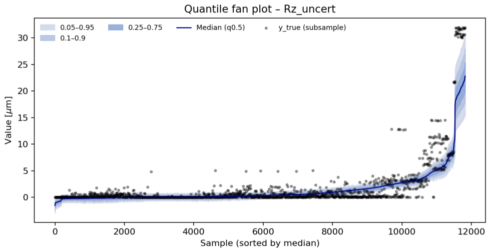

Figure S78: fan Rz uncert ()

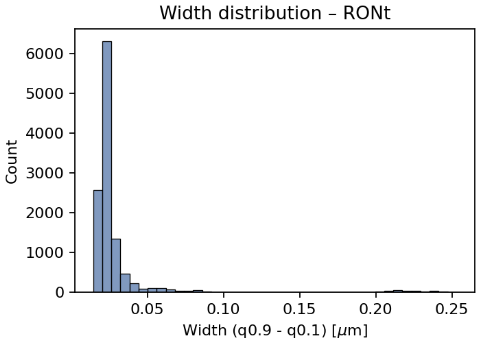

Figure S79: width RONt ()

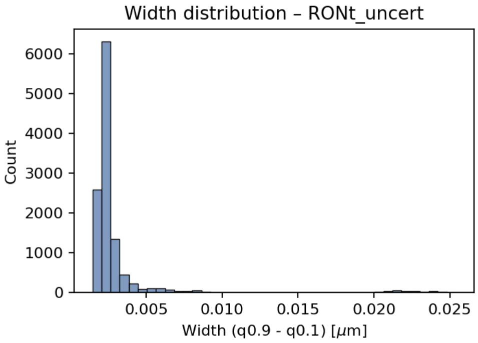

Figure S80: width RONt uncert ()

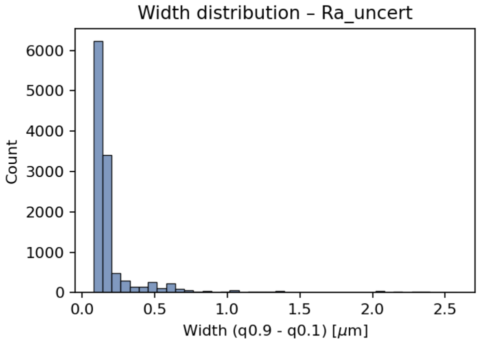

Figure S81: width Ra uncert ()

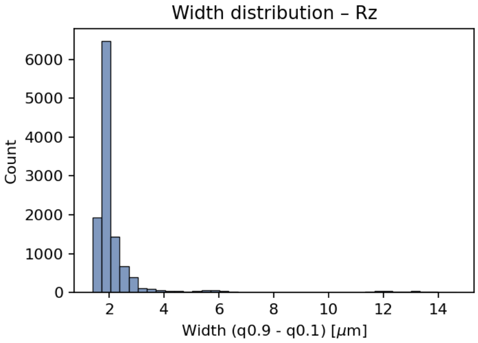

Figure S82: width Rz ()

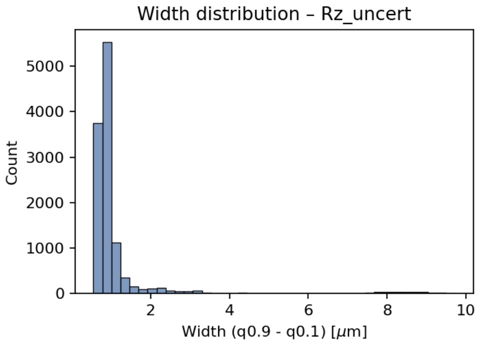

Figure S83: width Rz uncert ()

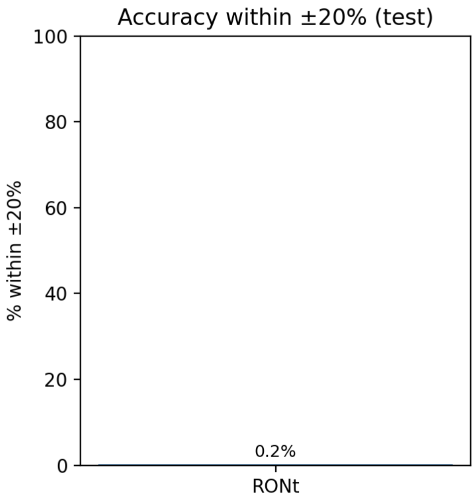

Figure S84: accuracy within tol 20percent ()

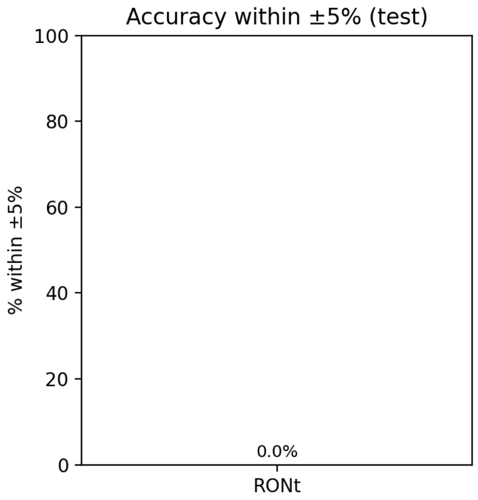

Figure S85: accuracy within tol 5percent ()

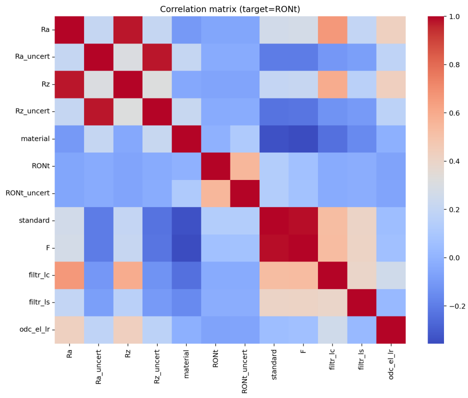

Figure S86: correlation heatmap RONt ()

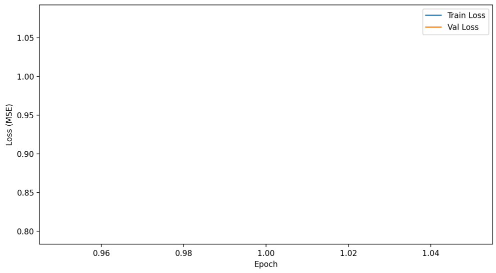

Figure S87: loss curves ()

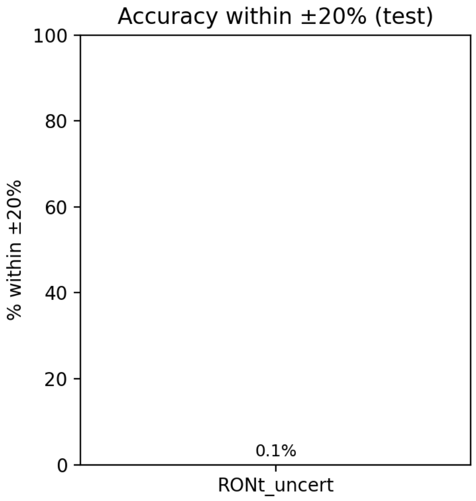

Figure S88: accuracy within tol 20percent ()

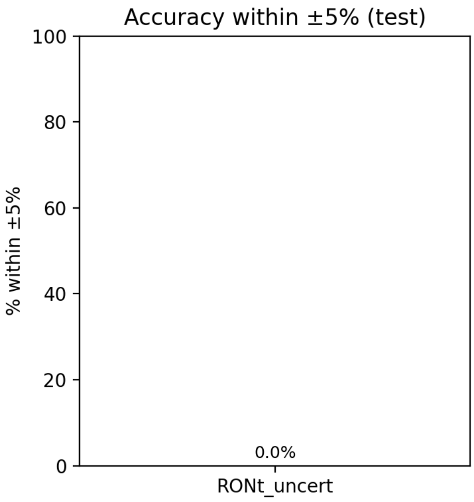

Figure S89: accuracy within tol 5percent ()

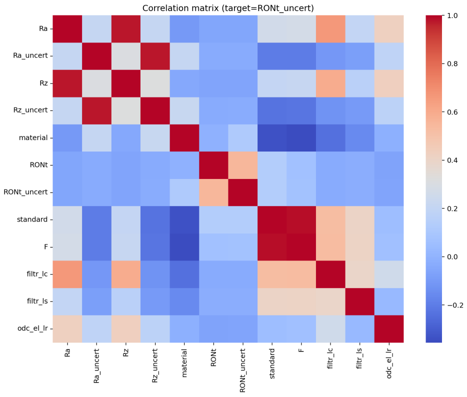

Figure S90: correlation heatmap RONt uncert ()

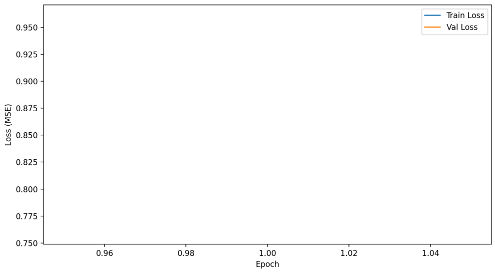

Figure S91: loss curves ()

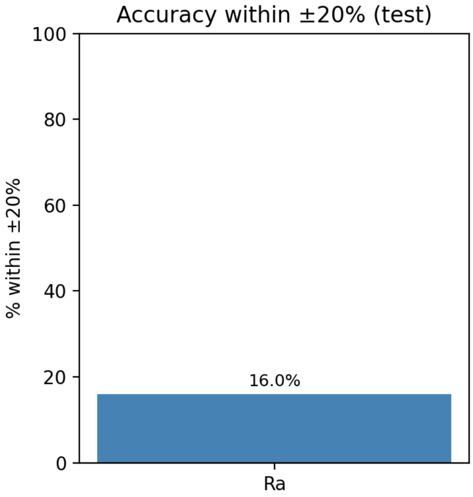

Figure S92: accuracy within tol 20percent ()

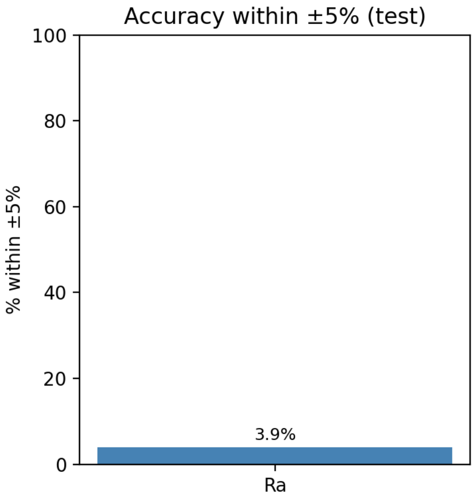

Figure S93: accuracy within tol 5percent ()

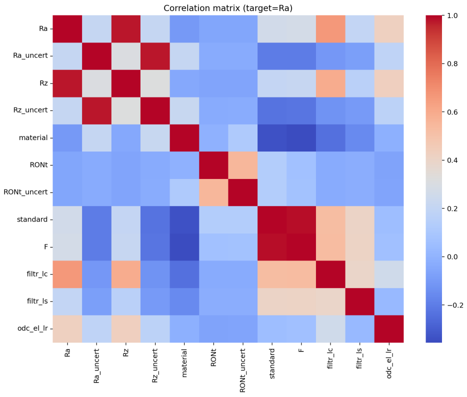

Figure S94: correlation heatmap Ra ()

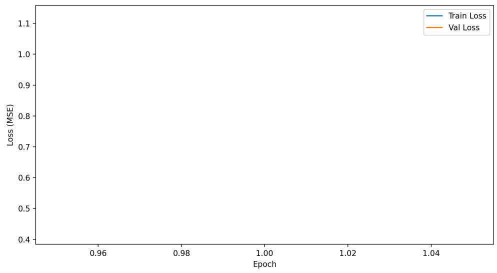

Figure S95: loss curves ()

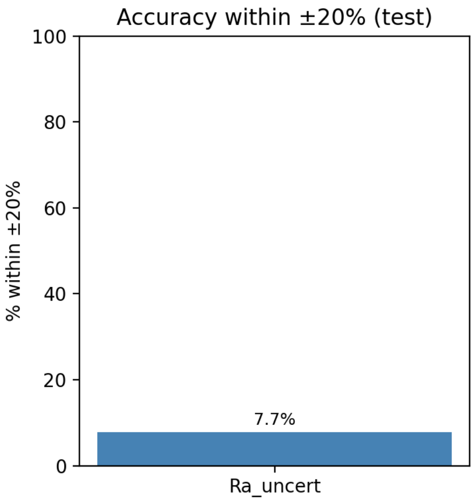

Figure S96: accuracy within tol 20percent ()

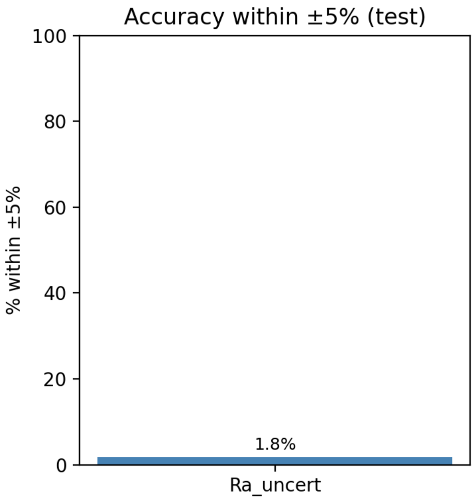

Figure S97: accuracy within tol 5percent ()

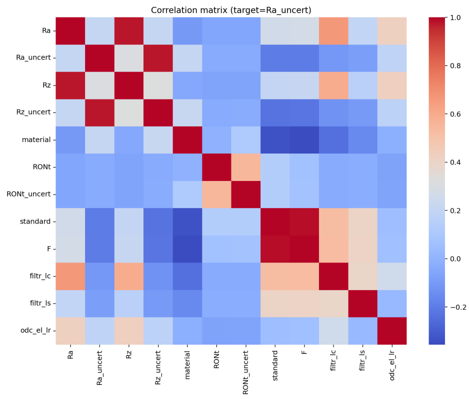

Figure S98: correlation heatmap Ra uncert ()

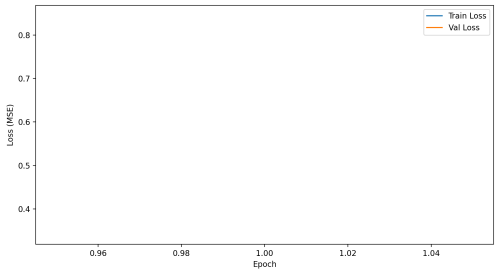

Figure S99: loss curves ()

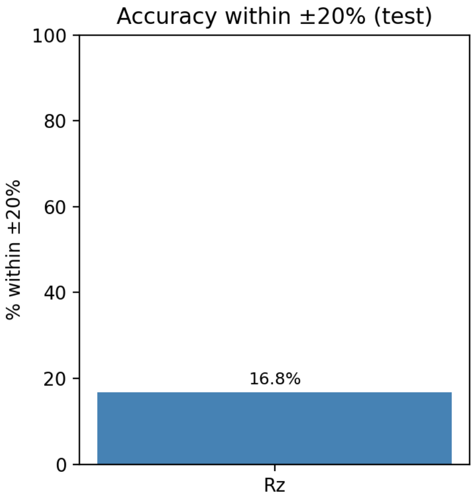

Figure S100: accuracy within tol 20percent ()

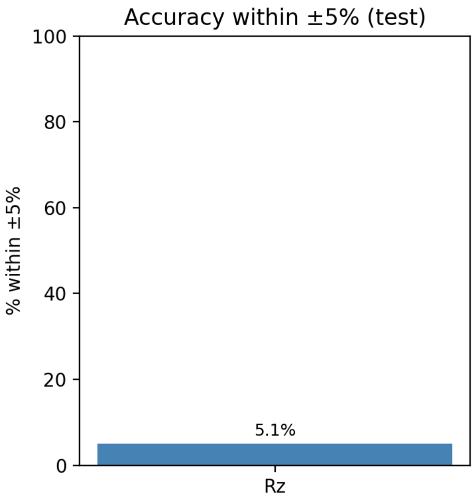

Figure S101: accuracy within tol 5percent ()

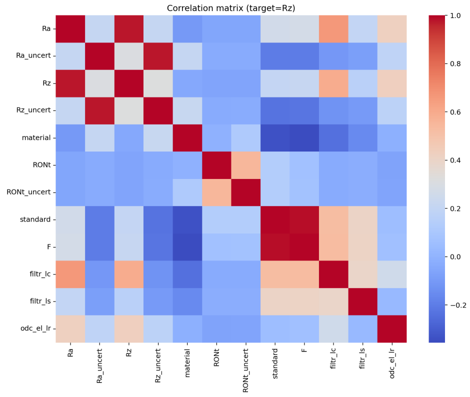

Figure S102: correlation heatmap Rz ()

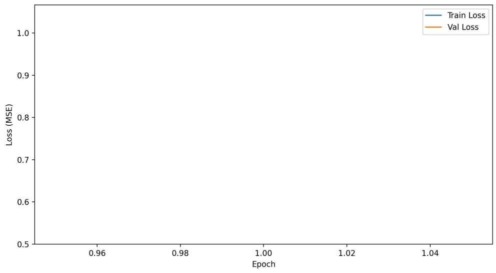

Figure S103: loss curves ()

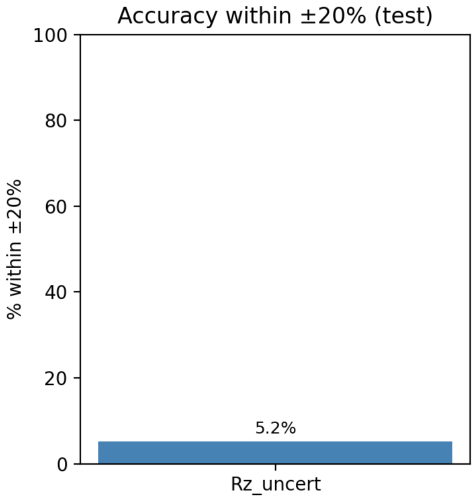

Figure S104: accuracy within tol 20percent ()

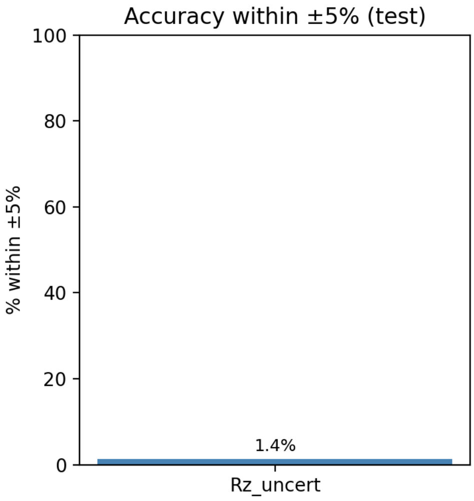

Figure S105: accuracy within tol 5percent ()

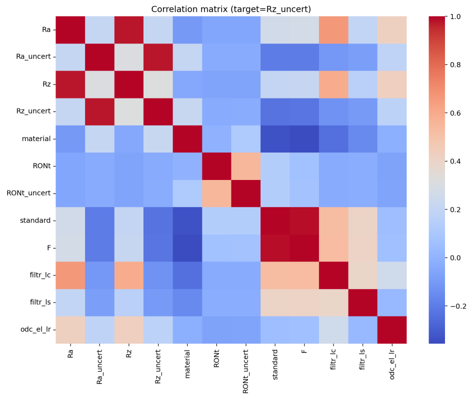

Figure S106: correlation heatmap Rz uncert ()

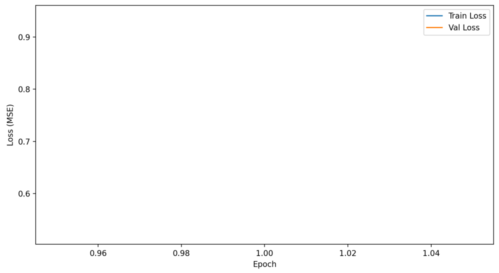

Figure S107: loss curves ()

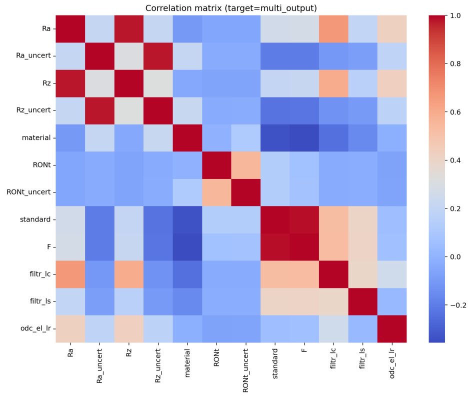

Figure S108: correlation heatmap multi output ()

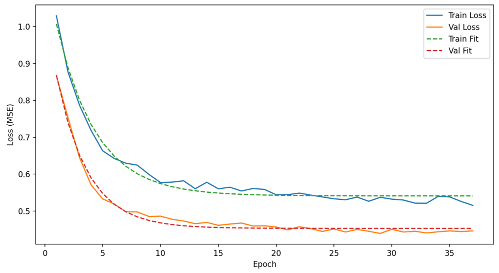

Figure S109: loss curves ()

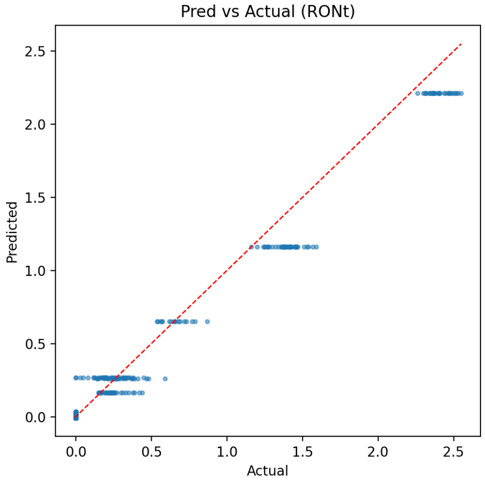

Figure S110: pred vs actual RONt ()

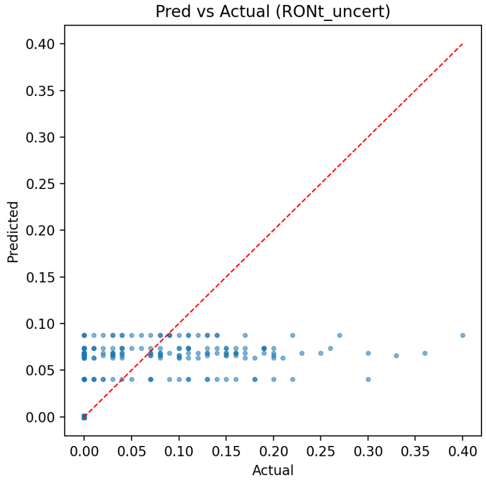

Figure S111: pred vs actual RONt uncert ()

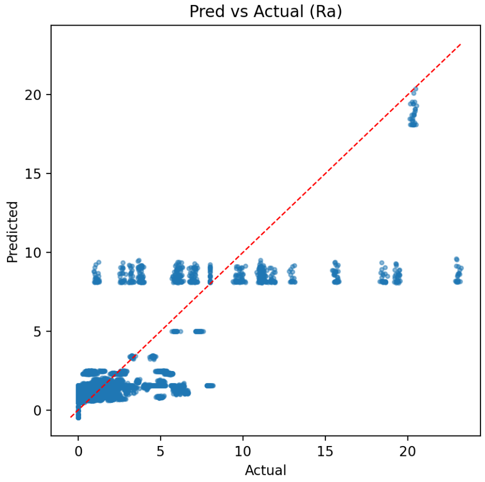

Figure S112: pred vs actual Ra ()

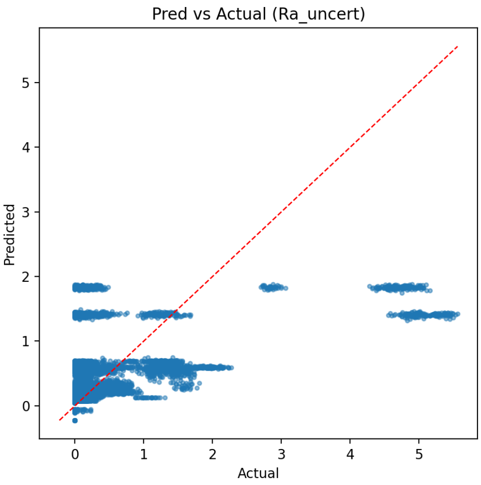

Figure S113: pred vs actual Ra uncert ()

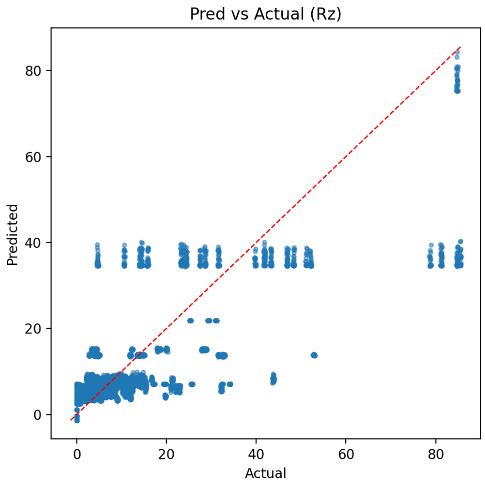

Figure S114: pred vs actual Rz ()

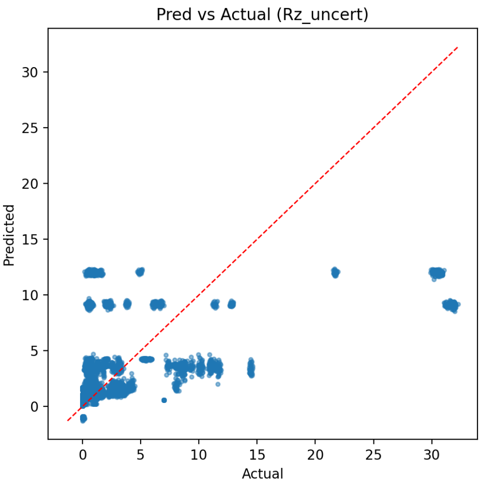

Figure S115: pred vs actual Rz uncert ()

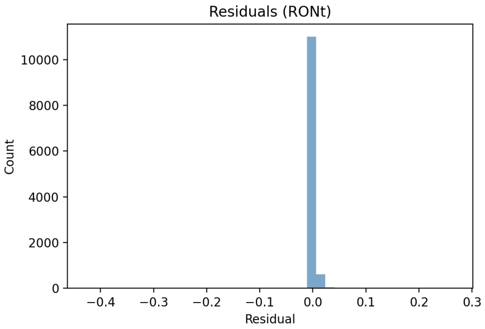

Figure S116: residuals hist RONt ()

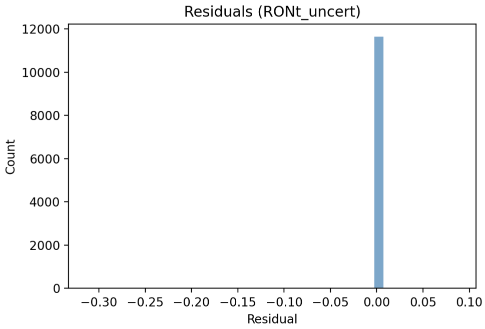

Figure S117: residuals hist RONt uncert ()

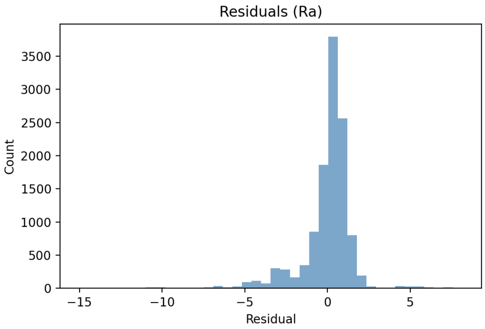

Figure S118: residuals hist Ra ()

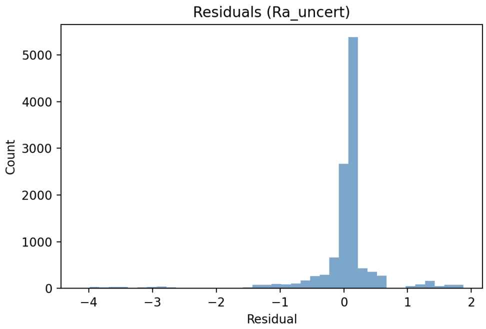

Figure S119: residuals hist Ra uncert ()

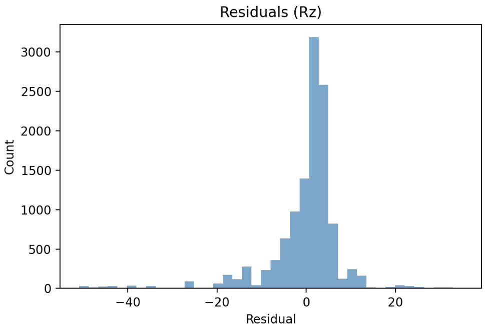

Figure S120: residuals hist Rz ()

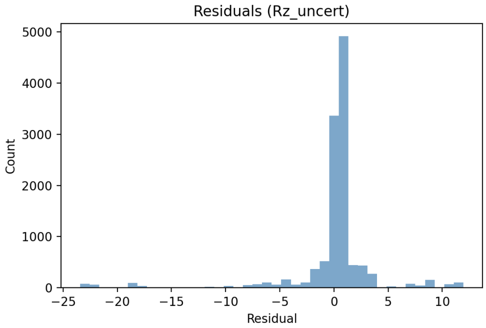

Figure S121: residuals hist Rz uncert ()

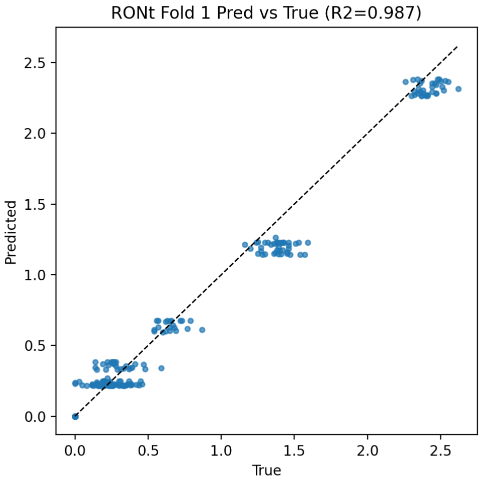

Figure S122: pred vs true ()

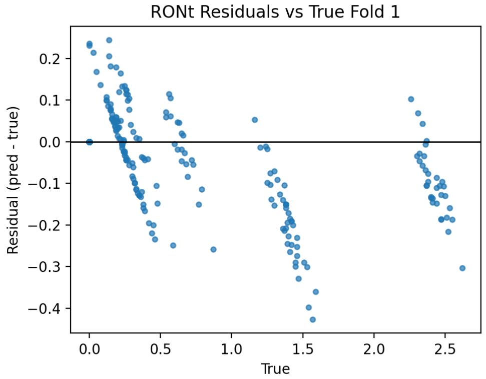

Figure S123: residuals vs true ()

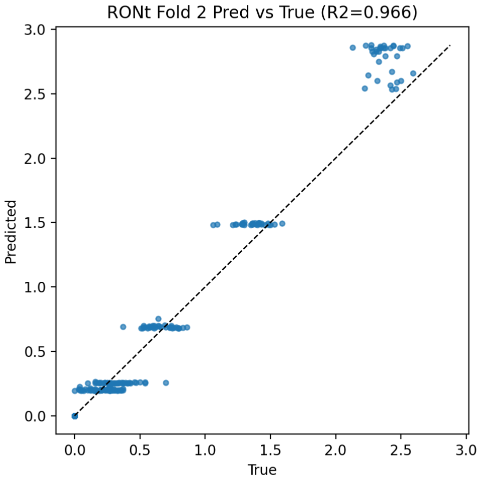

Figure S124: pred vs true ()

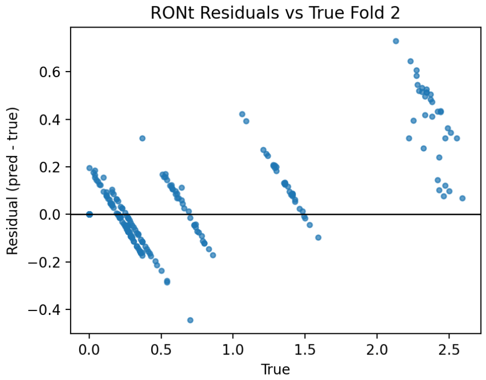

Figure S125: residuals vs true ()

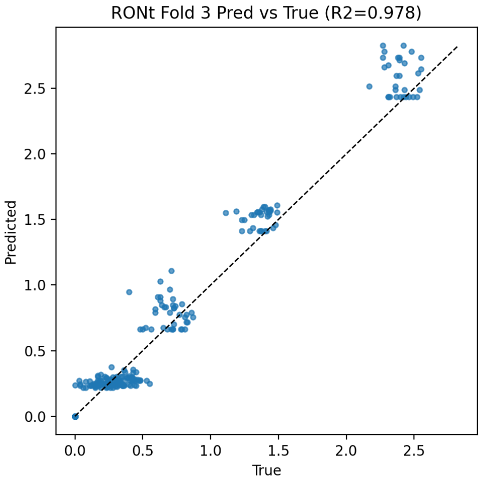

Figure S126: pred vs true ()

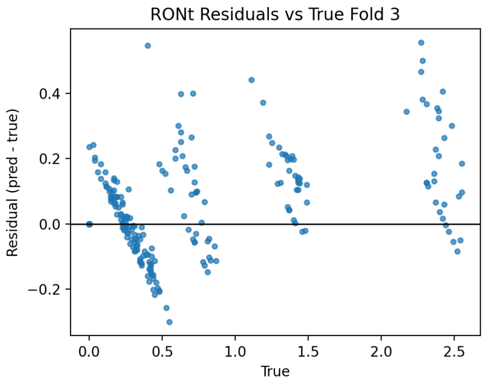

Figure S127: residuals vs true ()

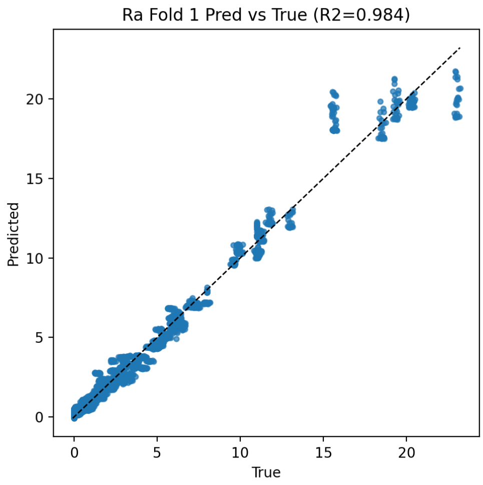

Figure S128: pred vs true ()

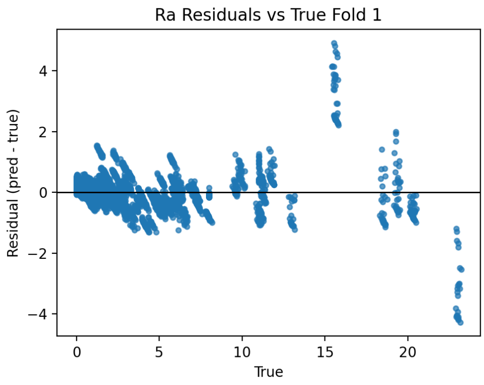

Figure S129: residuals vs true ()

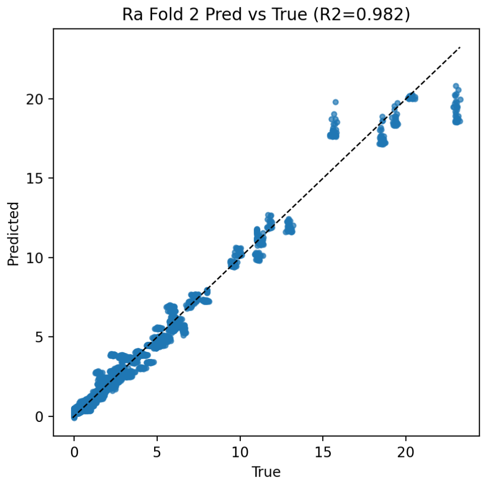

Figure S130: pred vs true ()

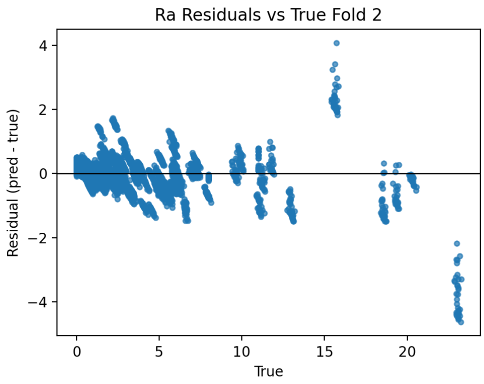

Figure S131: residuals vs true ()

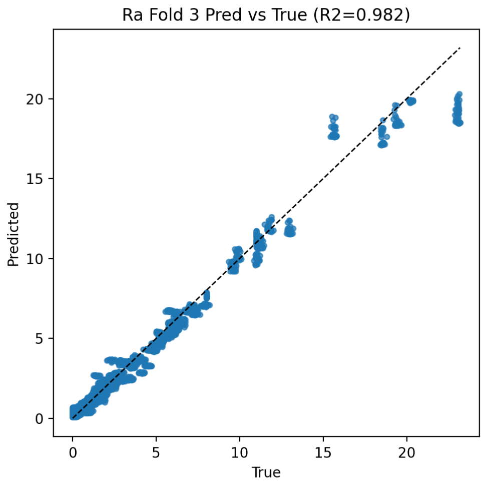

Figure S132: pred vs true ()

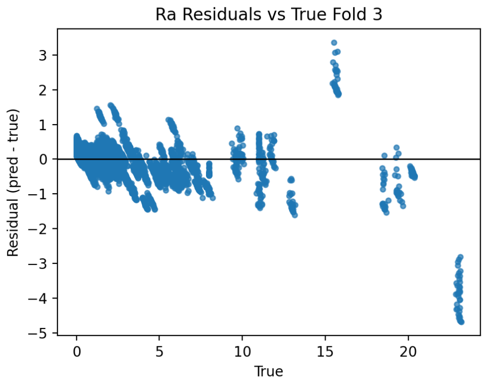

Figure S133: residuals vs true ()

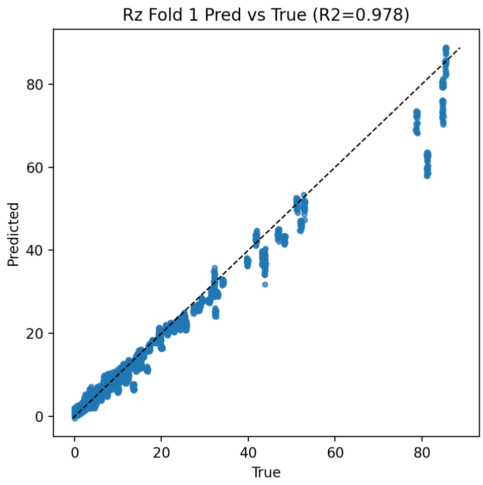

Figure S134: pred vs true ()

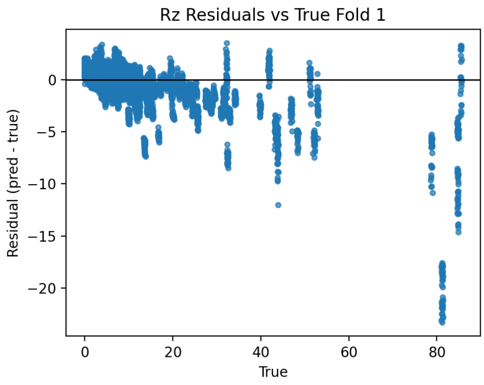

Figure S135: residuals vs true ()

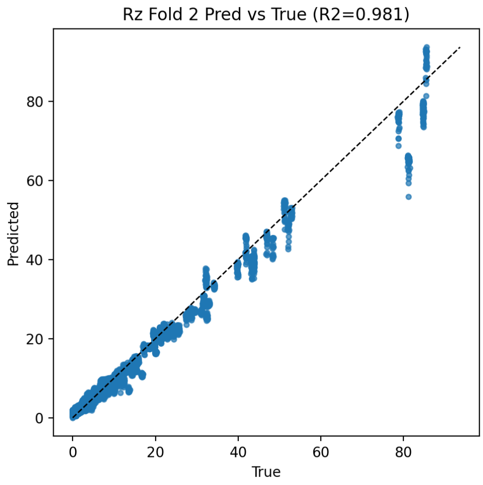

Figure S136: pred vs true ()

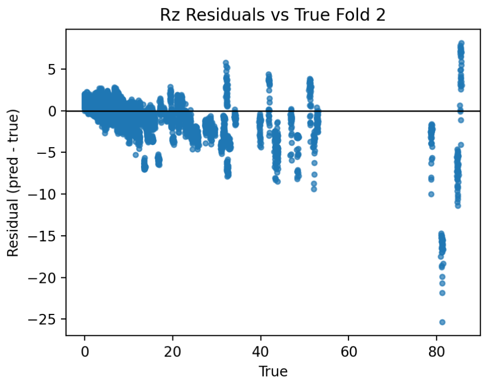

Figure S137: residuals vs true ()

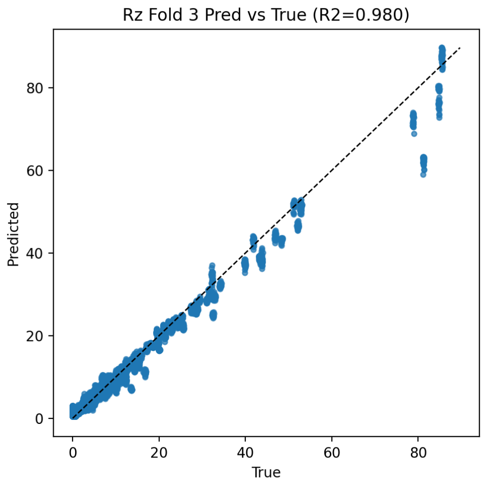

Figure S138: pred vs true ()

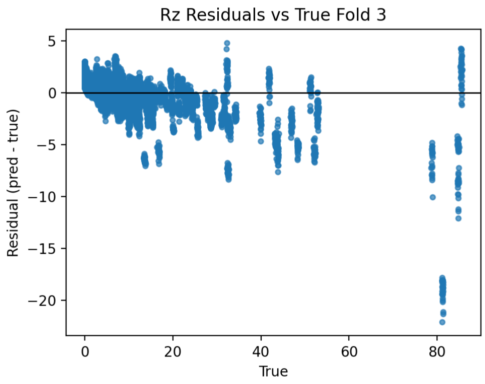

Figure S139: residuals vs true ()

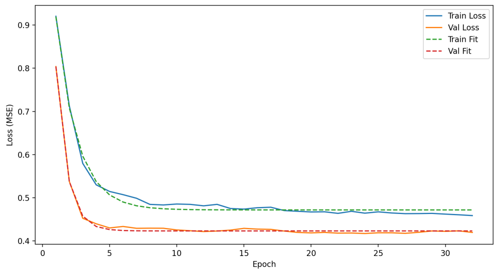

Figure S140: loss curves ()

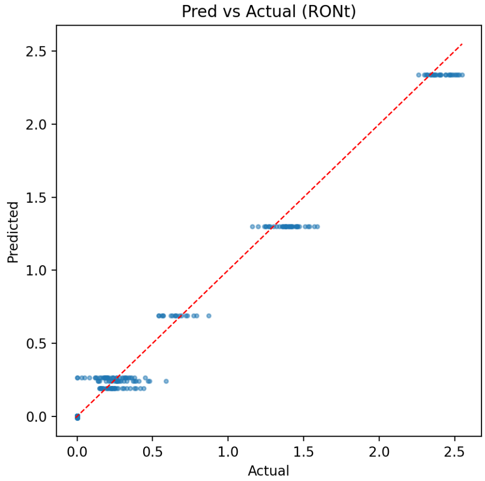

Figure S141: pred vs actual RONt ()

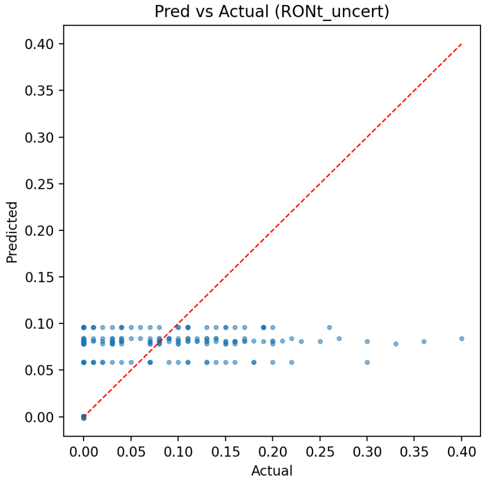

Figure S142: pred vs actual RONt uncert ()

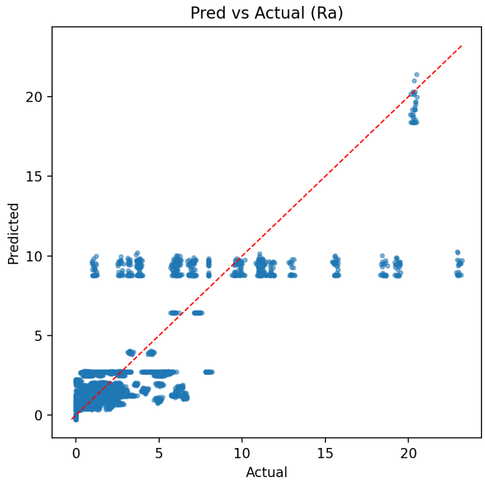

Figure S143: pred vs actual Ra ()

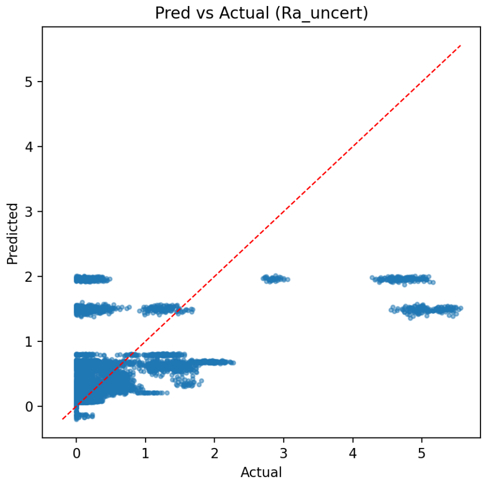

Figure S144: pred vs actual Ra uncert ()

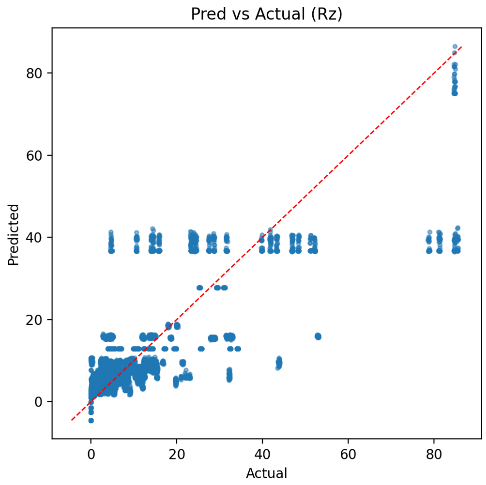

Figure S145: pred vs actual Rz ()

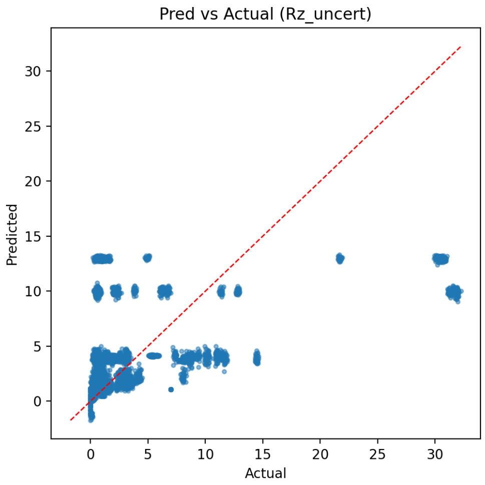

Figure S146: pred vs actual Rz uncert ()

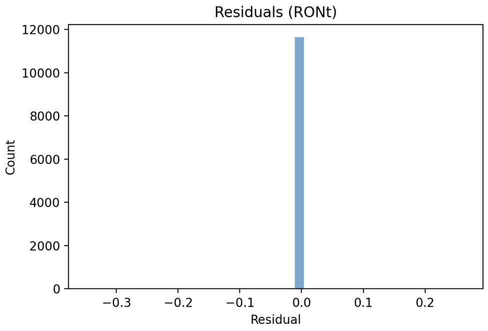

Figure S147: residuals hist RONt ()

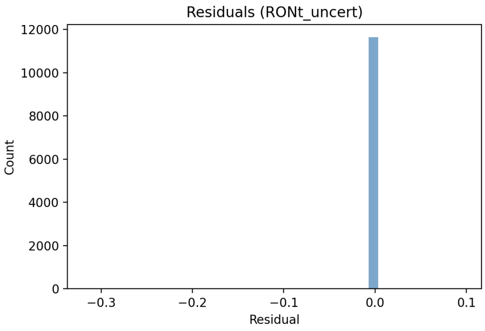

Figure S148: residuals hist RONt uncert ()

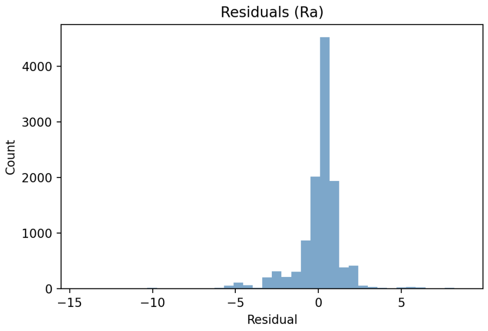

Figure S149: residuals hist Ra ()

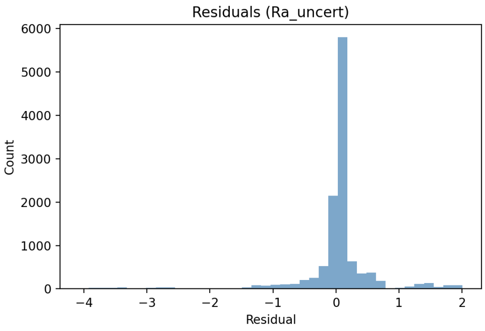

Figure S150: residuals hist Ra uncert ()

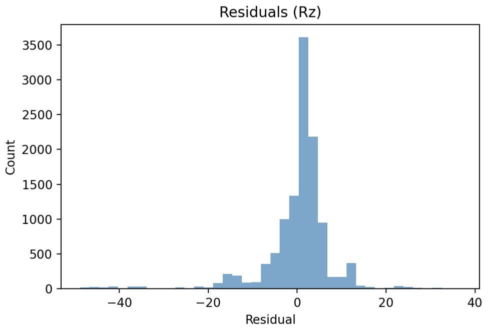

Figure S151: residuals hist Rz ()

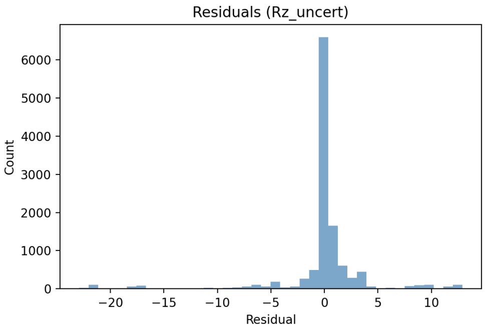

Figure S152: residuals hist Rz uncert ()

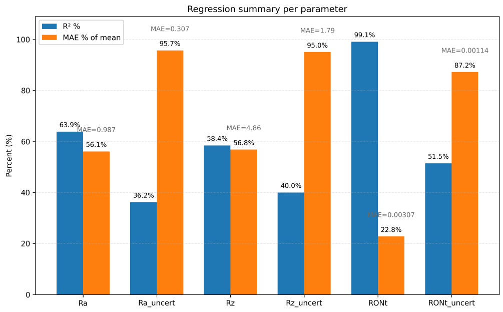

Figure S153: regression summary bars ()

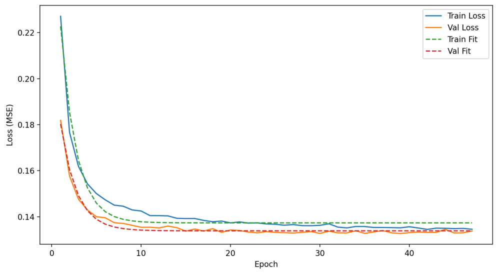

Figure S154: loss curves ()

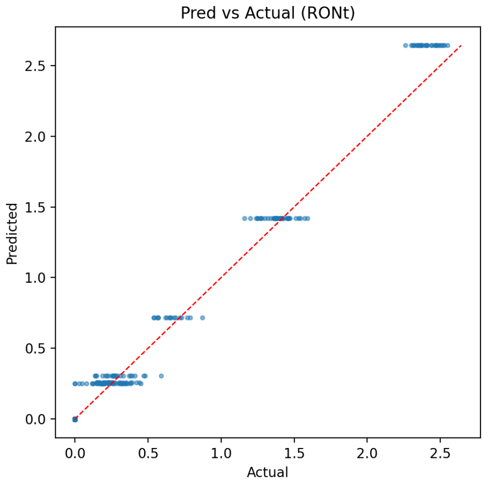

Figure S155: pred vs actual RONt ()

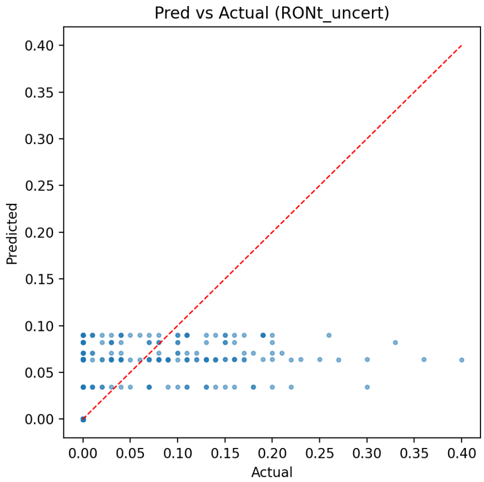

Figure S156: pred vs actual RONt uncert ()

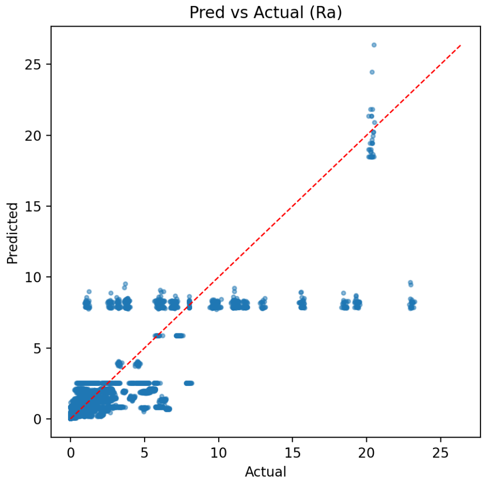

Figure S157: pred vs actual Ra ()

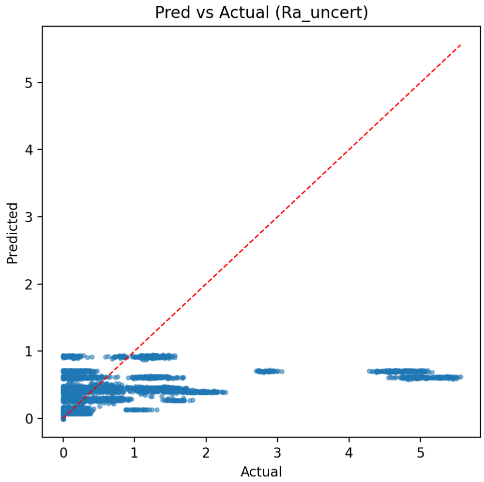

Figure S158: pred vs actual Ra uncert ()

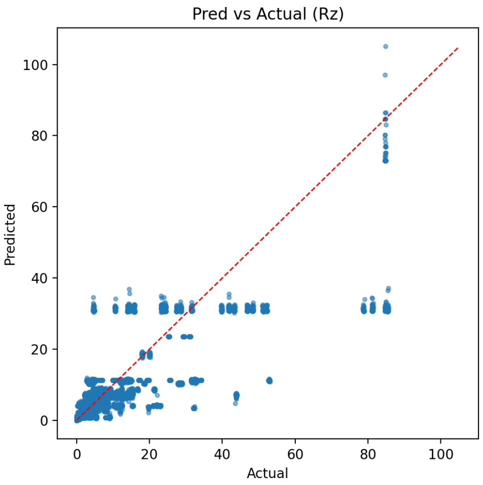

Figure S159: pred vs actual Rz ()

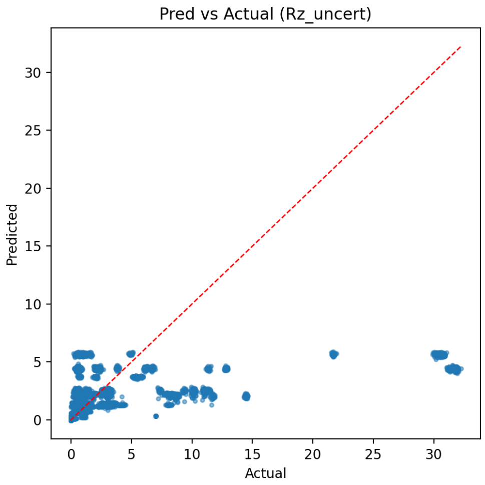

Figure S160: pred vs actual Rz uncert ()

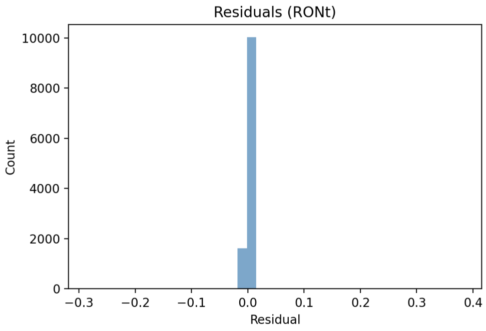

Figure S161: residuals hist RONt ()

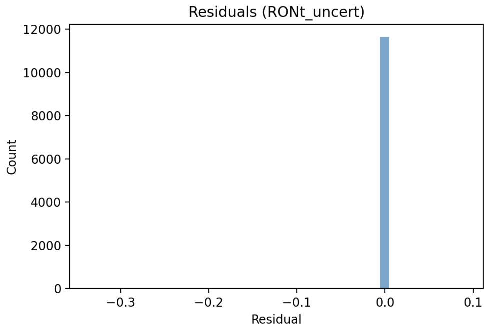

Figure S162: residuals hist RONt uncert ()

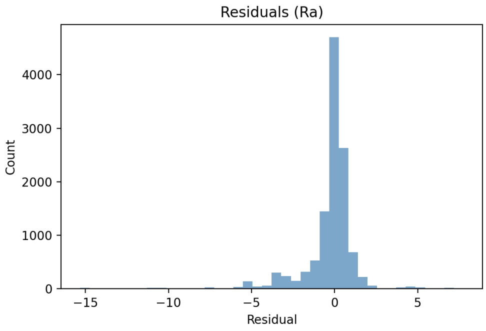

Figure S163: residuals hist Ra ()

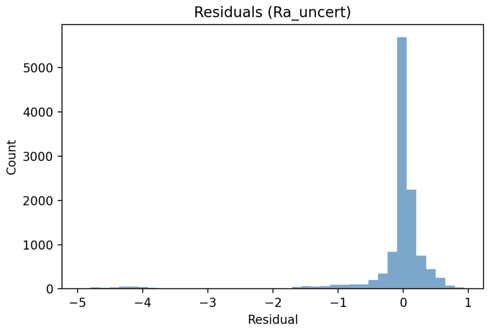

Figure S164: residuals hist Ra uncert ()

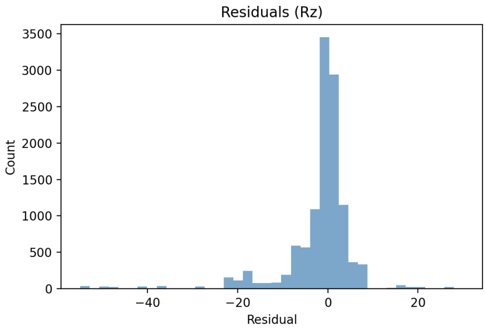

Figure S165: residuals hist Rz ()

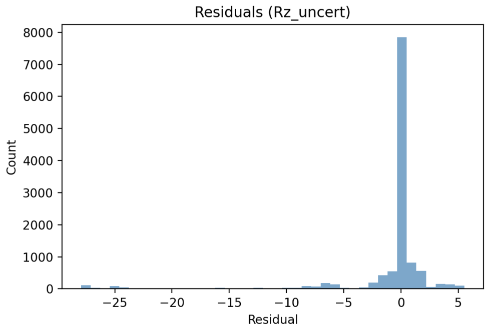

Figure S166: residuals hist Rz uncert ()

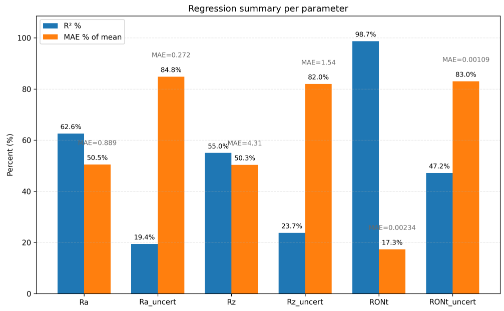

Figure S167: regression summary bars ()

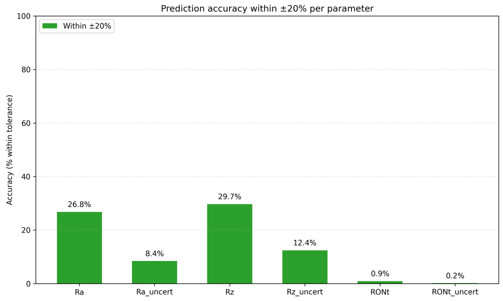

Figure S168: accuracy within tol 20percent ()

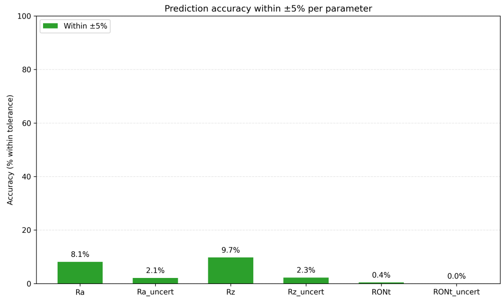

Figure S169: accuracy within tol 5percent ()

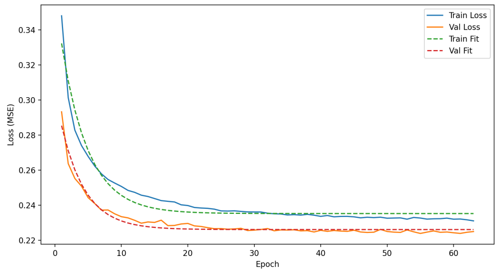

Figure S170: loss curves ()

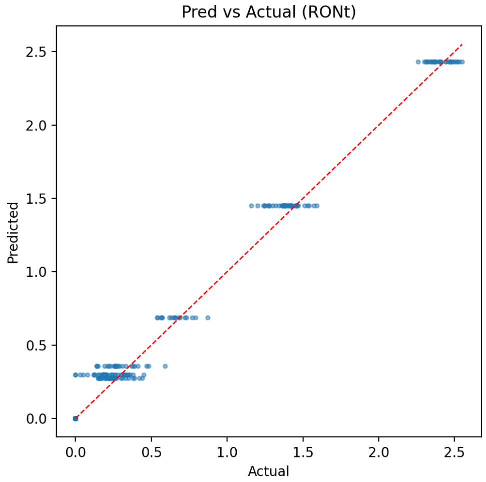

Figure S171: pred vs actual RONt ()

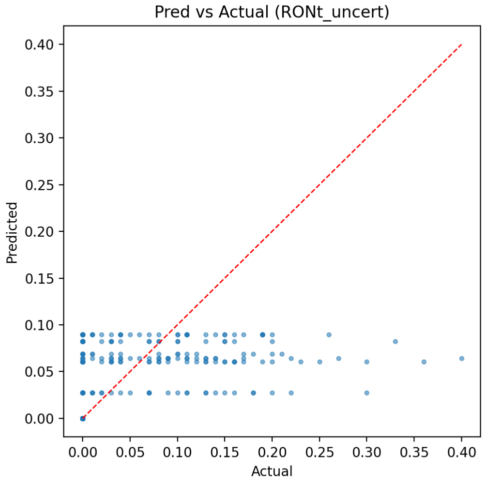

Figure S172: pred vs actual RONt uncert ()

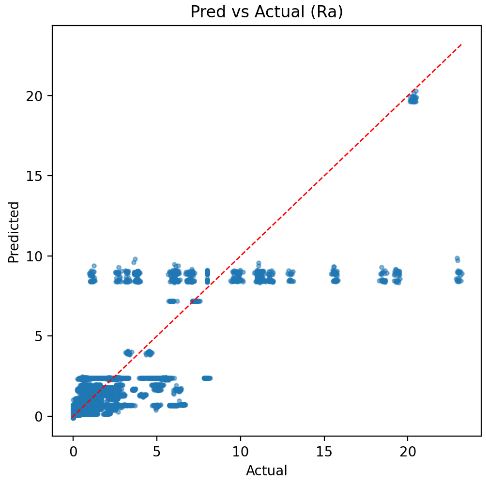

Figure S173: pred vs actual Ra ()

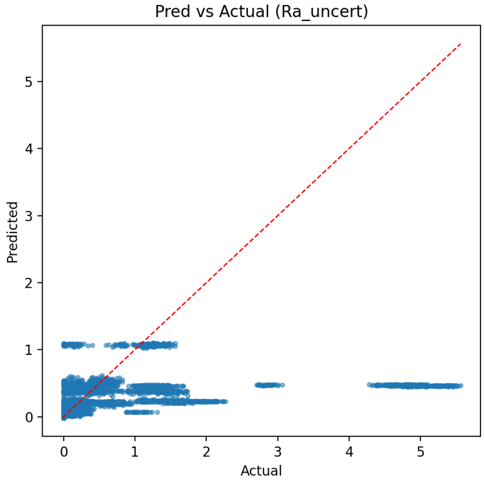

Figure S174: pred vs actual Ra uncert ()

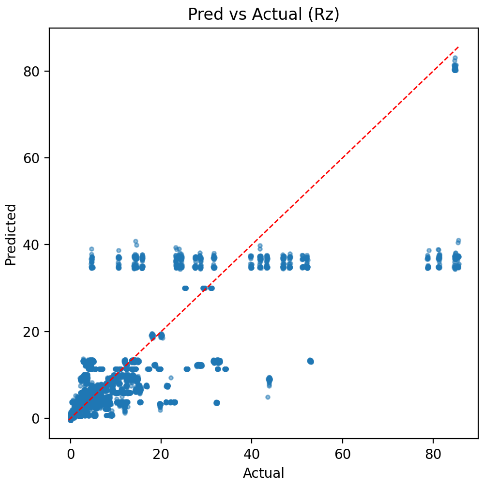

Figure S175: pred vs actual Rz ()

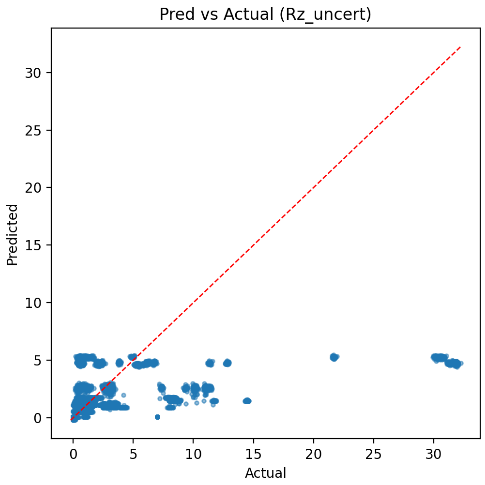

Figure S176: pred vs actual Rz uncert ()

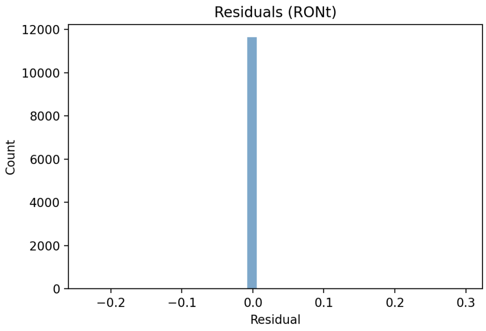

Figure S177: residuals hist RONt ()

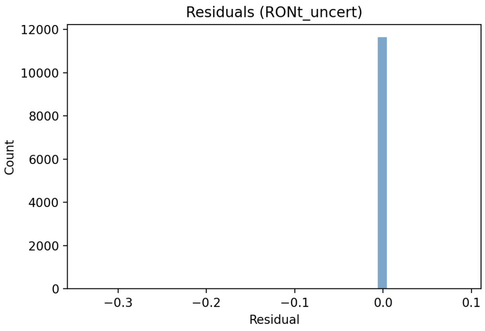

Figure S178: residuals hist RONt uncert ()

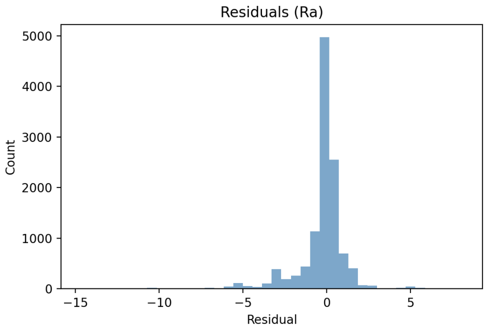

Figure S179: residuals hist Ra ()

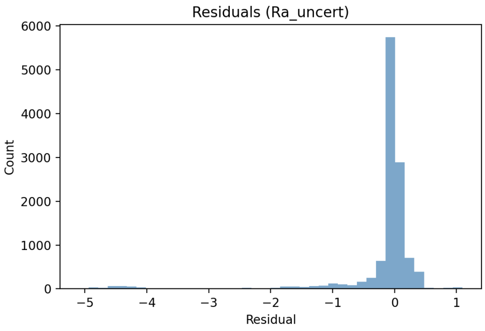

Figure S180: residuals hist Ra uncert ()

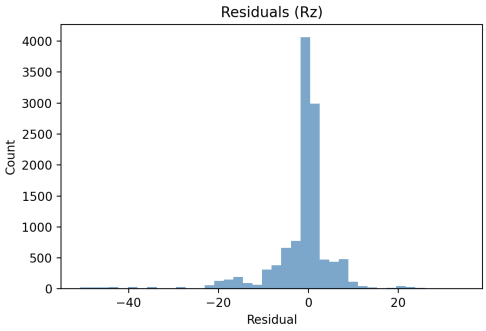

Figure S181: residuals hist Rz ()

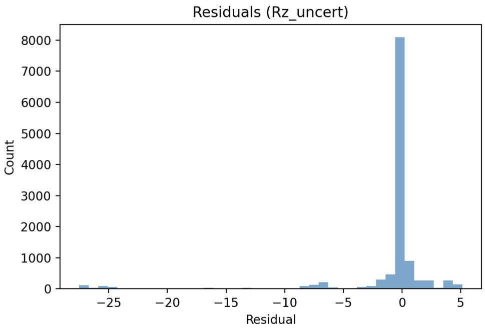

Figure S182: residuals hist Rz uncert ()

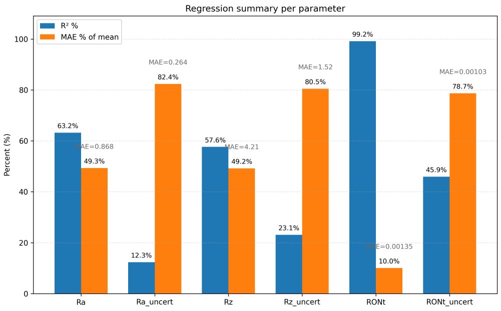

Figure S183: regression summary bars ()

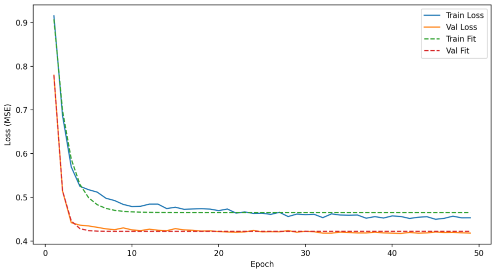

Figure S184: loss curves ()

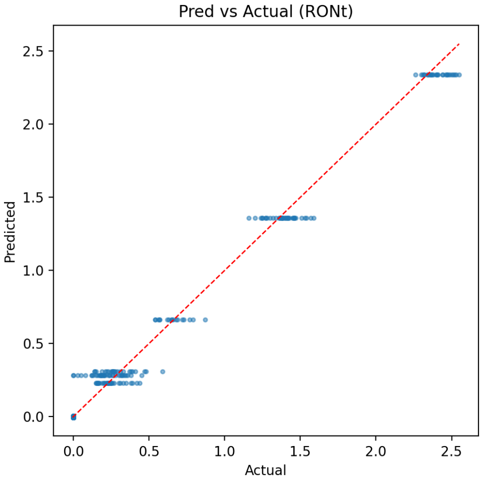

Figure S185: pred vs actual RONt ()

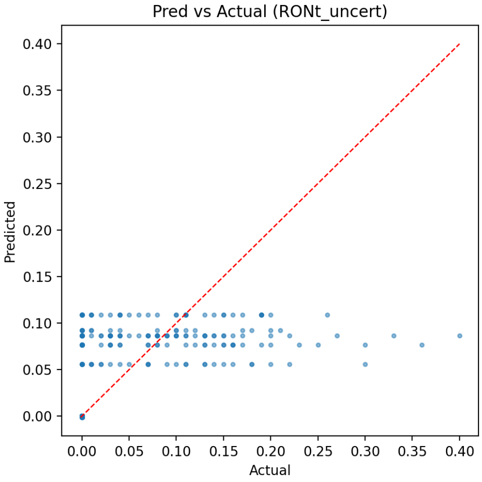

Figure S186: pred vs actual RONt uncert ()

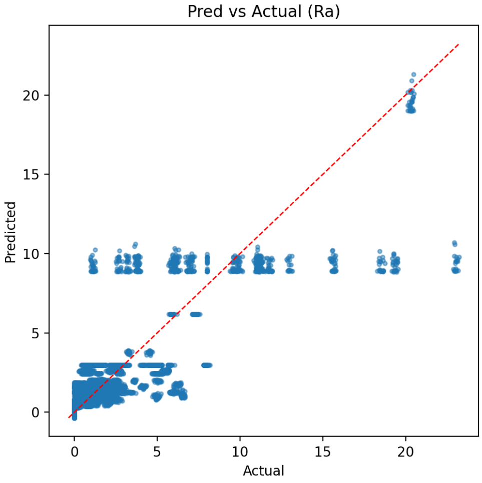

Figure S187: pred vs actual Ra ()

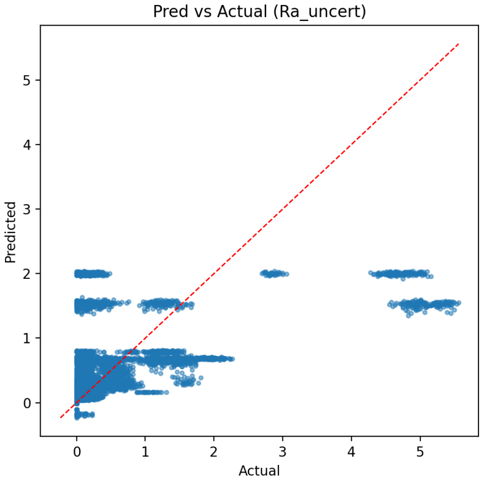

Figure S188: pred vs actual Ra uncert ()

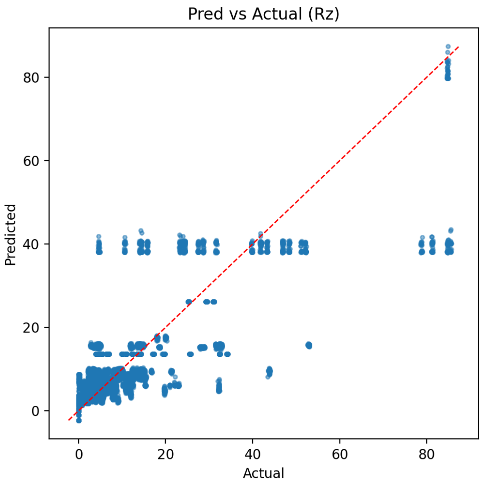

Figure S189: pred vs actual Rz ()

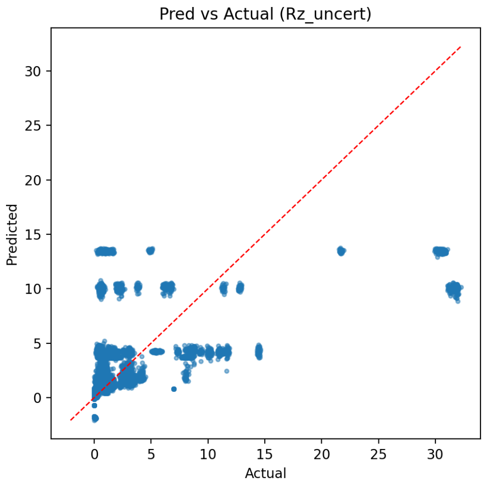

Figure S190: pred vs actual Rz uncert ()

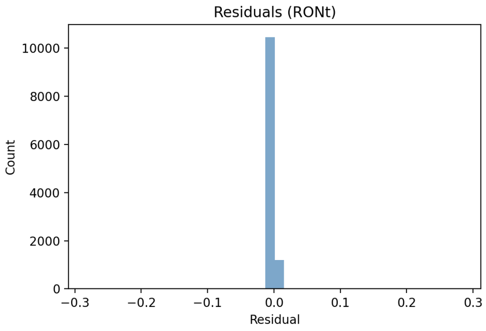

Figure S191: residuals hist RONt ()

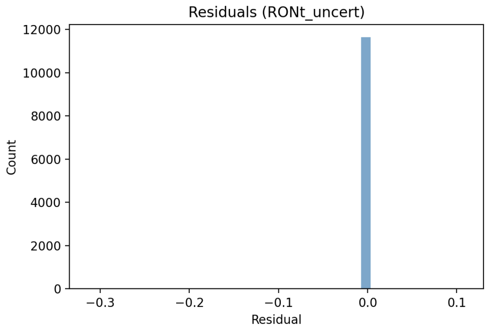

Figure S192: residuals hist RONt uncert ()

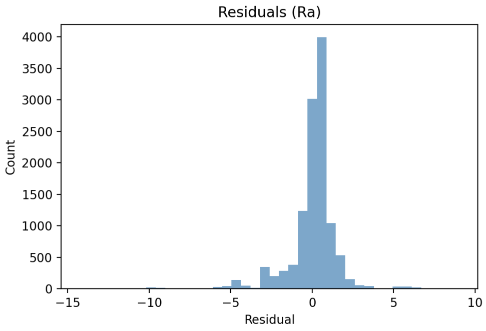

Figure S193: residuals hist Ra ()

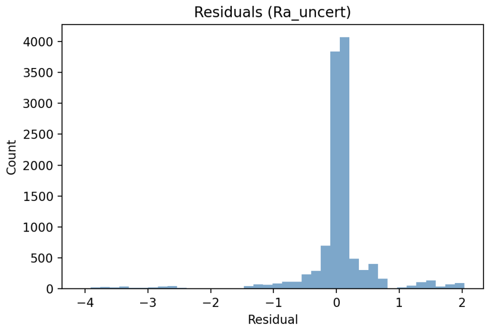

Figure S194: residuals hist Ra uncert ()

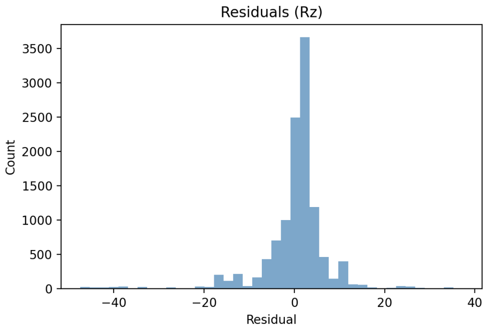

Figure S195: residuals hist Rz ()

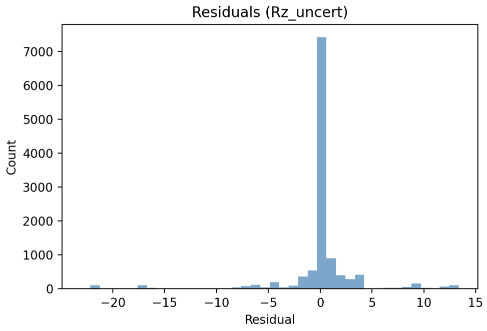

Figure S196: residuals hist Rz uncert ()

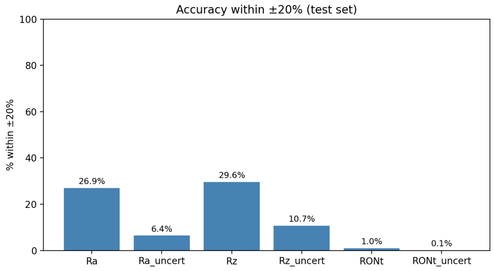

Figure S197: accuracy within tol 20percent ()

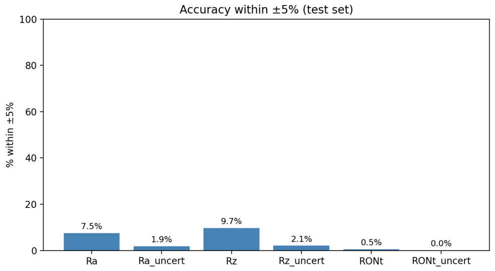

Figure S198: accuracy within tol 5percent ()

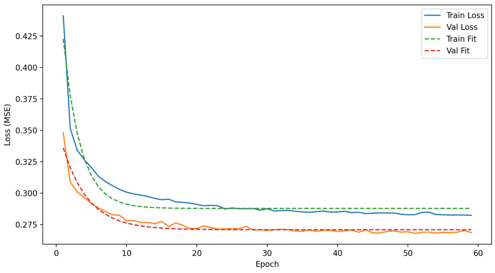

Figure S199: loss curves ()

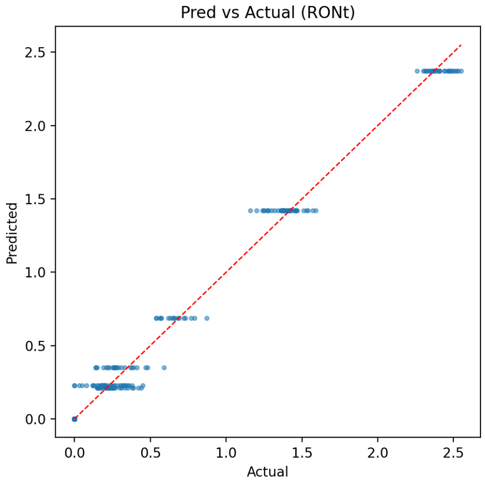

Figure S200: pred vs actual RONt ()

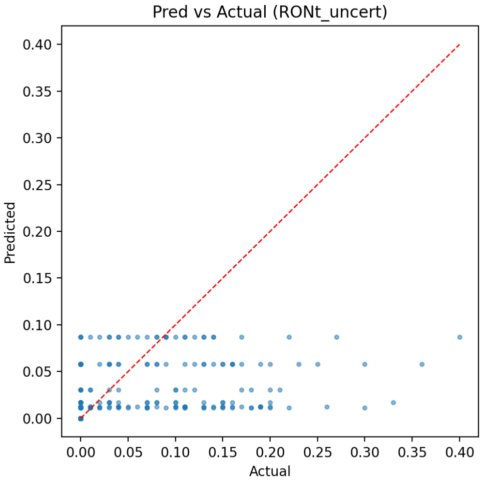

Figure S201: pred vs actual RONt uncert ()

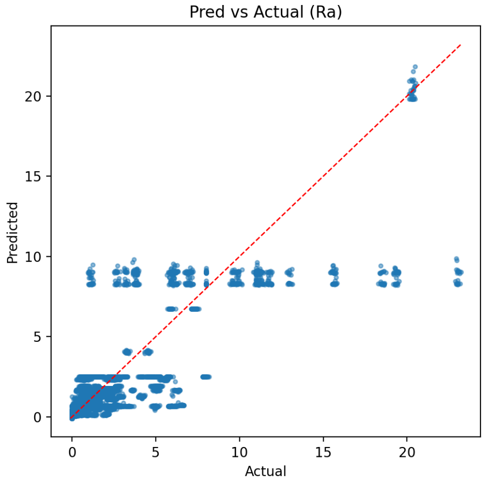

Figure S202: pred vs actual Ra ()

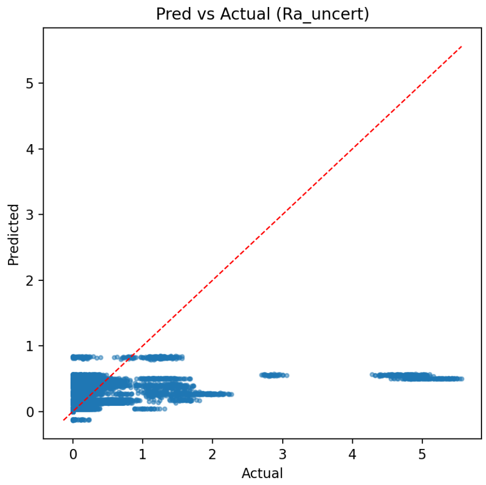

Figure S203: pred vs actual Ra uncert ()

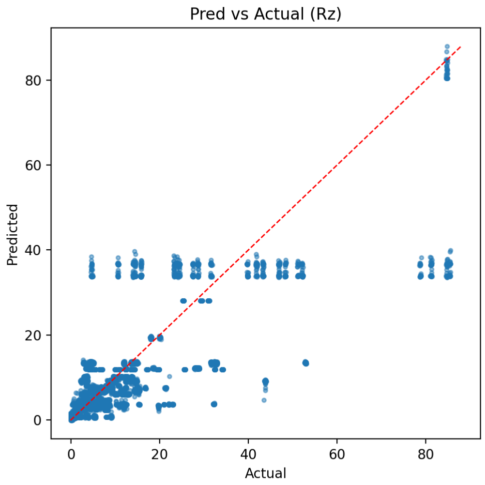

Figure S204: pred vs actual Rz ()

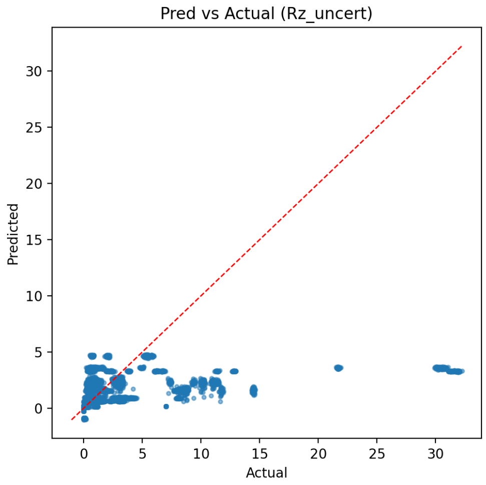

Figure S205: pred vs actual Rz uncert ()

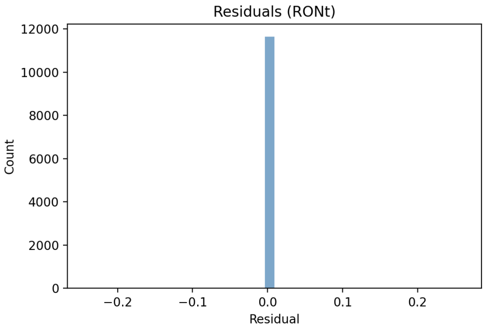

Figure S206: residuals hist RONt ()

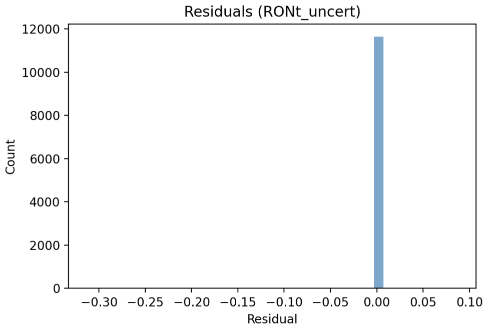

Figure S207: residuals hist RONt uncert ()

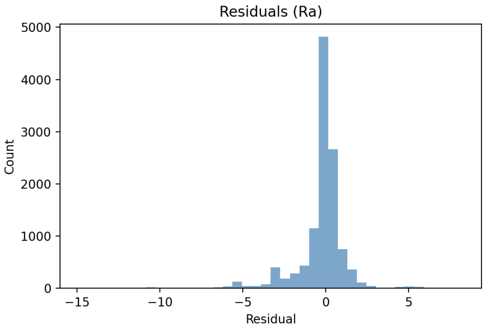

Figure S208: residuals hist Ra ()

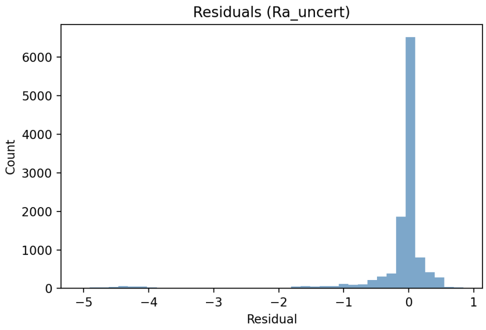

Figure S209: residuals hist Ra uncert ()

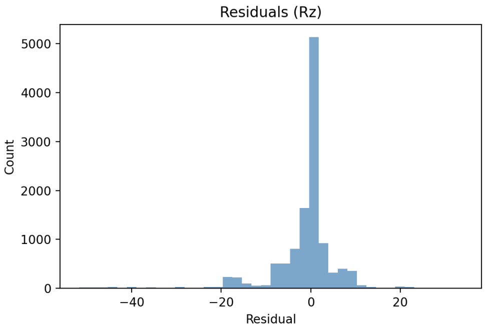

Figure S210: residuals hist Rz ()

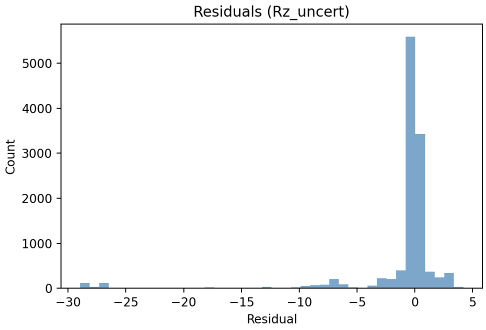

Figure S211: residuals hist Rz uncert ()

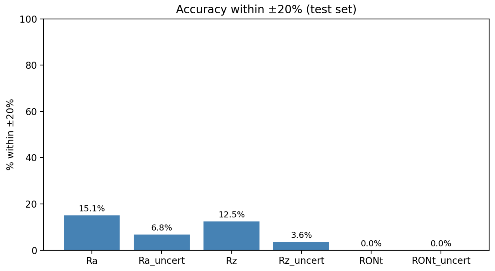

Figure S212: accuracy within tol 20percent ()

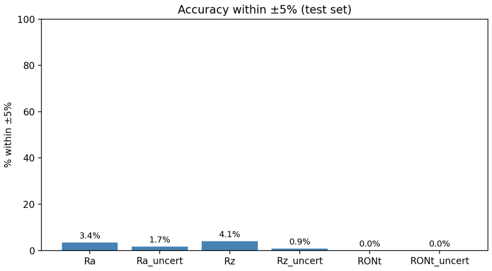

Figure S213: accuracy within tol 5percent ()

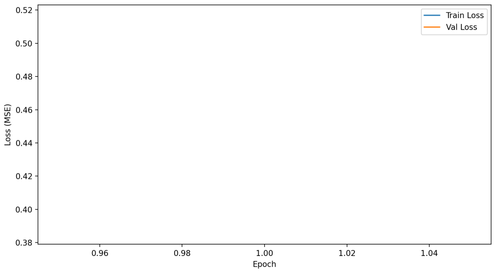

Figure S214: loss curves ()

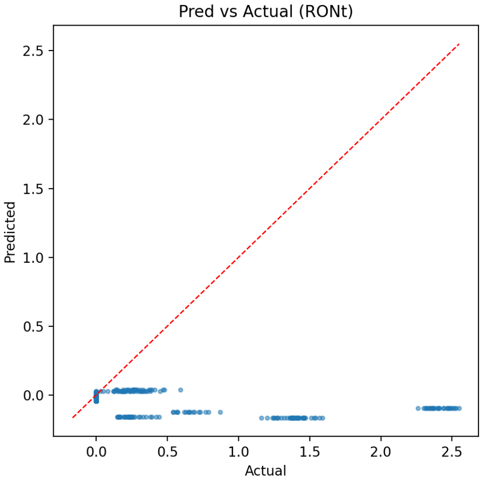

Figure S215: pred vs actual RONt ()

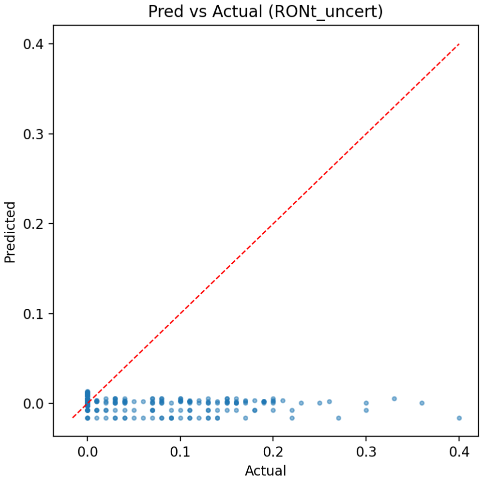

Figure S216: pred vs actual RONt uncert ()

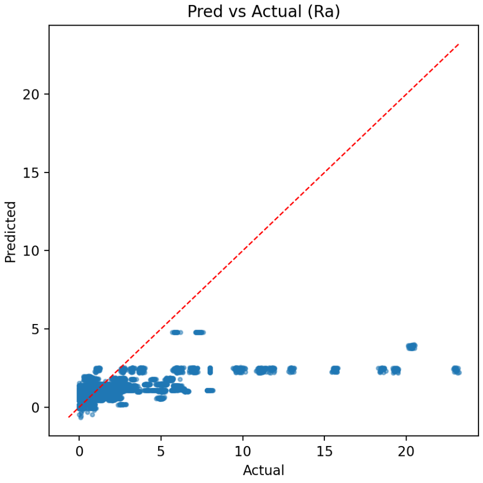

Figure S217: pred vs actual Ra ()

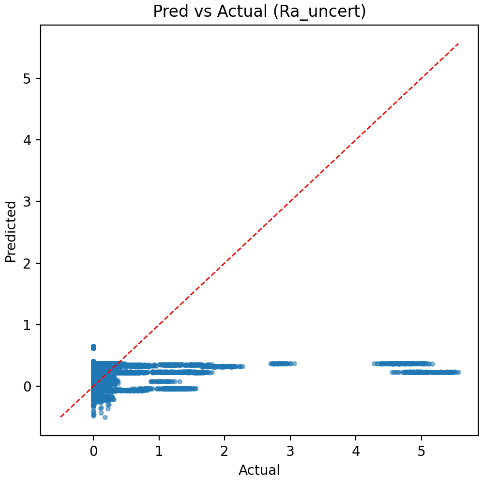

Figure S218: pred vs actual Ra uncert ()

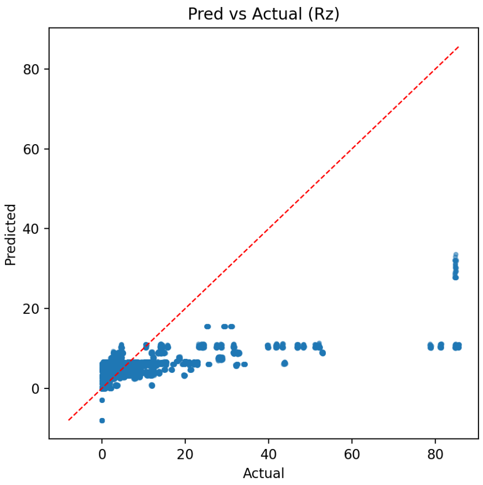

Figure S219: pred vs actual Rz ()

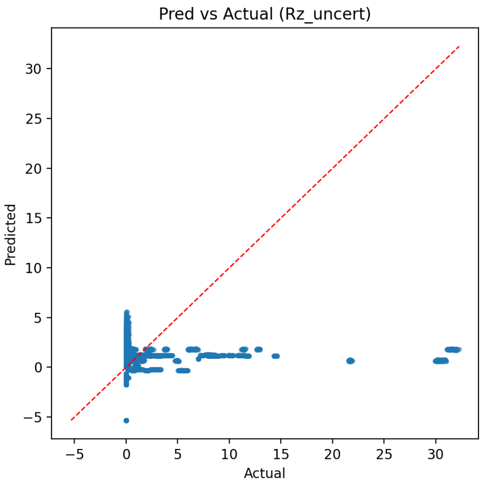

Figure S220: pred vs actual Rz uncert ()

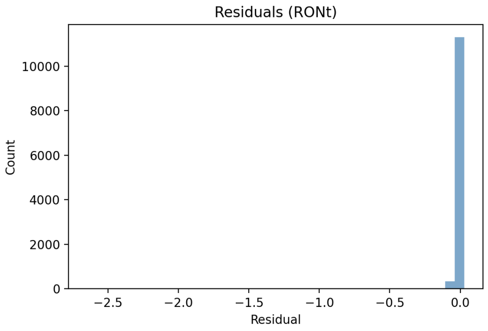

Figure S221: residuals hist RONt ()

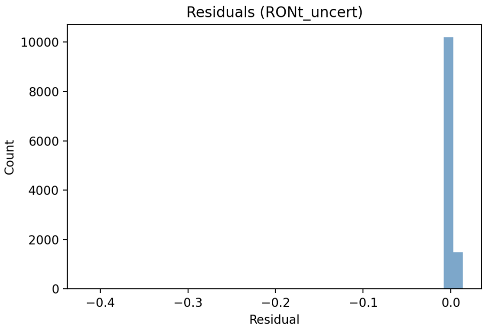

Figure S222: residuals hist RONt uncert ()

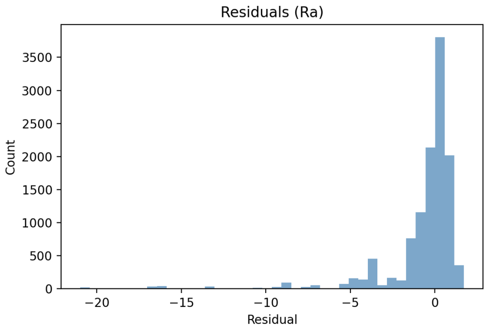

Figure S223: residuals hist Ra ()

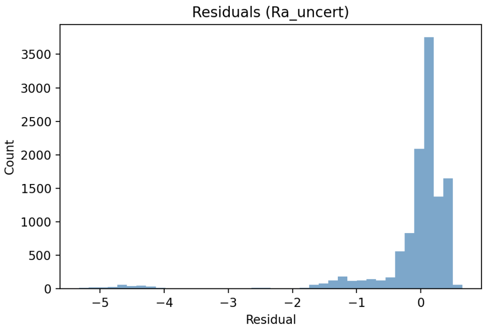

Figure S224: residuals hist Ra uncert ()

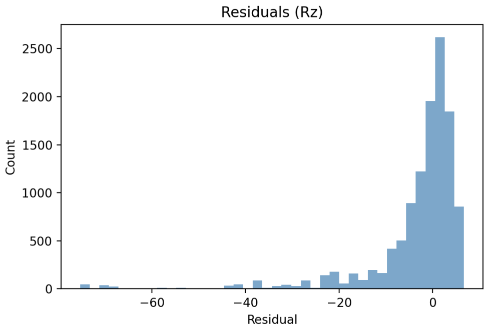

Figure S225: residuals hist Rz ()

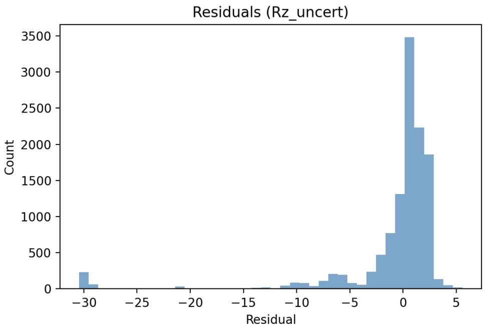

Figure S226: residuals hist Rz uncert ()

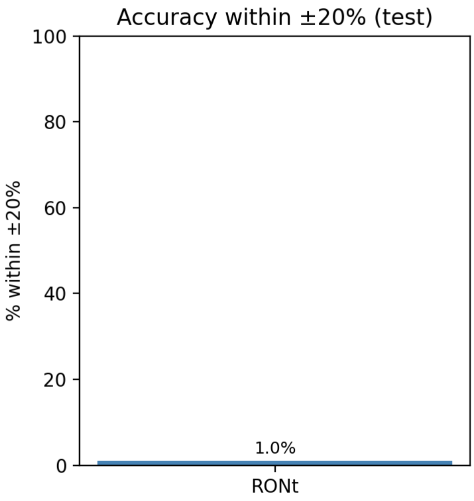

Figure S227: accuracy within tol 20percent ()

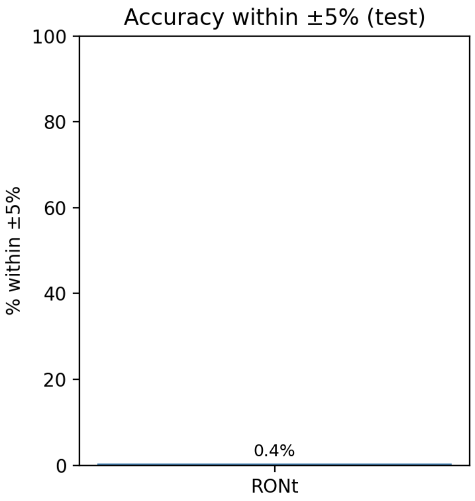

Figure S228: accuracy within tol 5percent ()

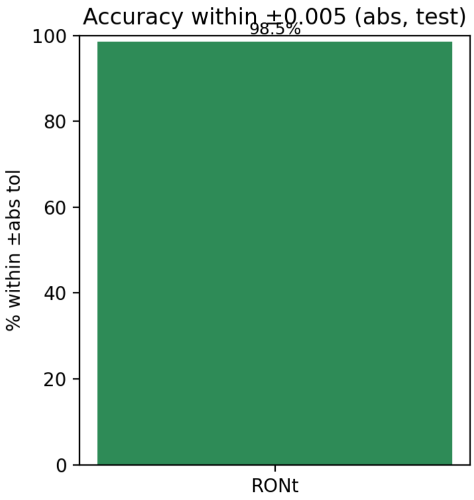

Figure S229: accuracy within tol abs 0p005 ()

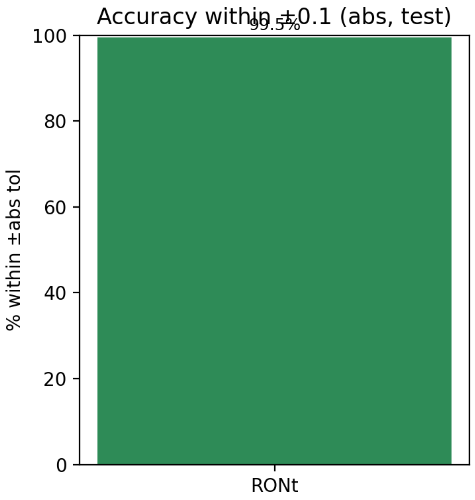

Figure S230: accuracy within tol abs 0p1 ()

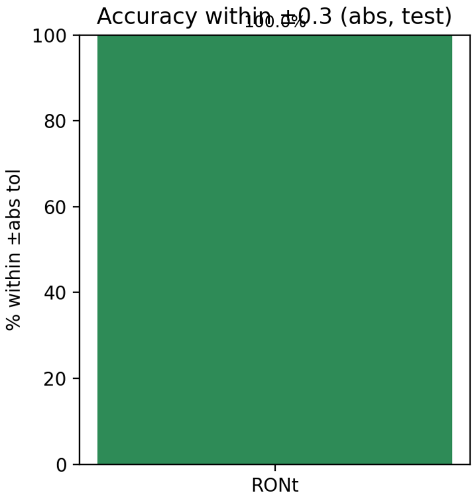

Figure S231: accuracy within tol abs 0p3 ()

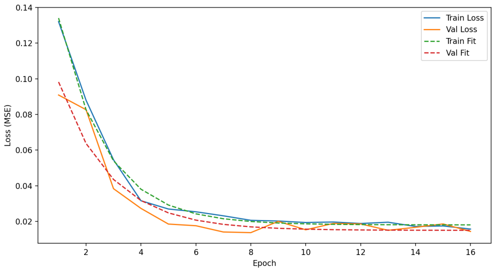

Figure S232: loss curves ()

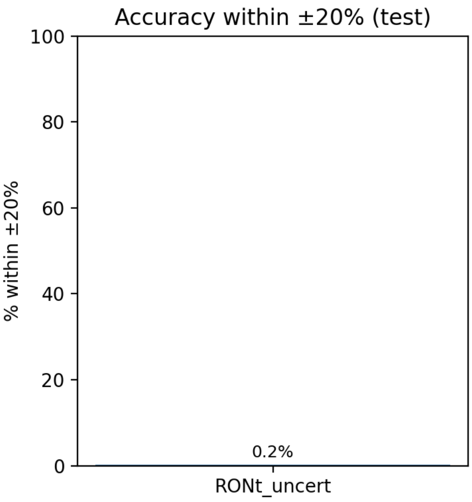

Figure S233: accuracy within tol 20percent ()

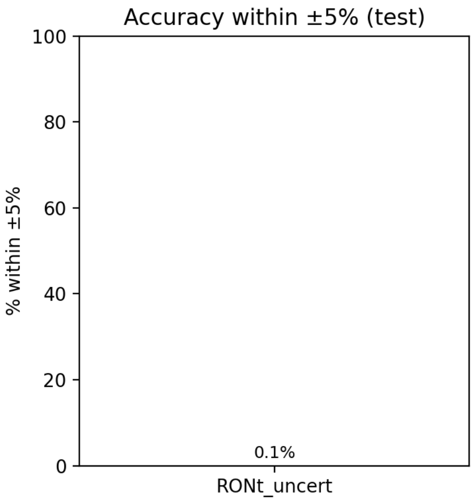

Figure S234: accuracy within tol 5percent ()

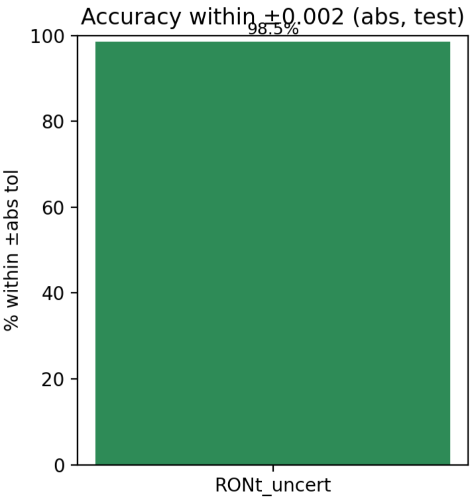

Figure S235: accuracy within tol abs 0p002 ()

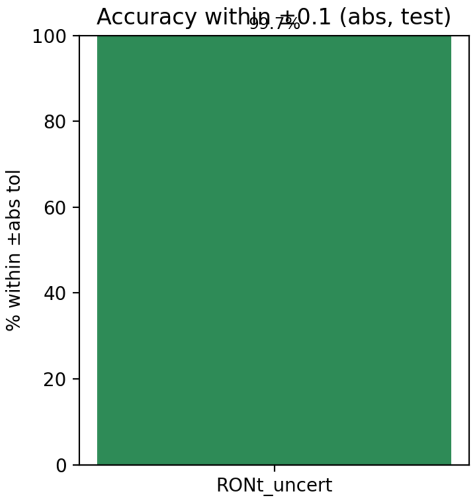

Figure S236: accuracy within tol abs 0p1 ()

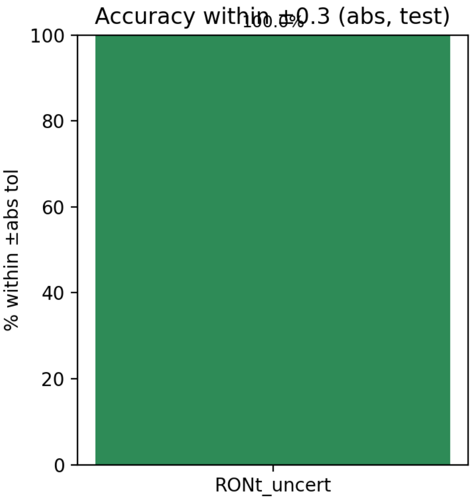

Figure S237: accuracy within tol abs 0p3 ()

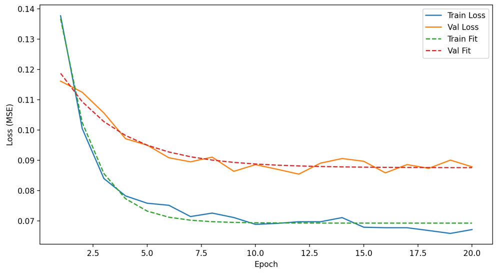

Figure S238: loss curves ()

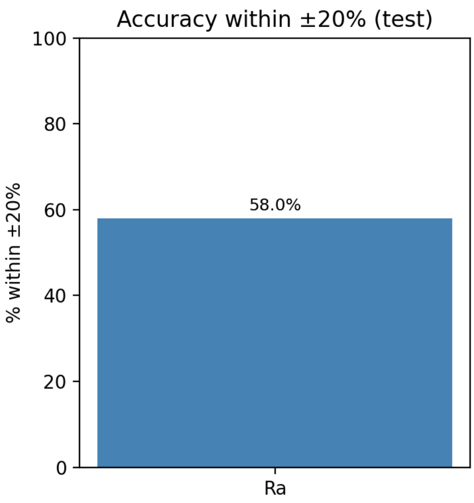

Figure S239: accuracy within tol 20percent ()

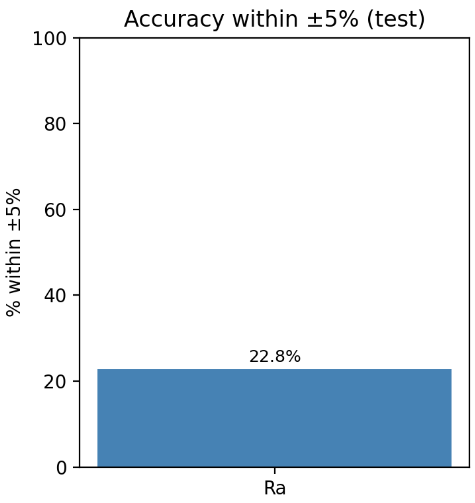

Figure S240: accuracy within tol 5percent ()

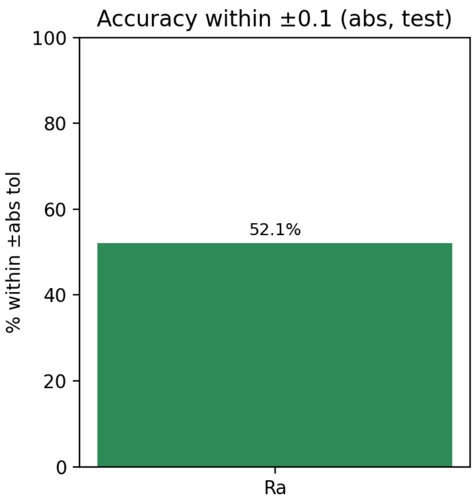

Figure S241: accuracy within tol abs 0p1 ()

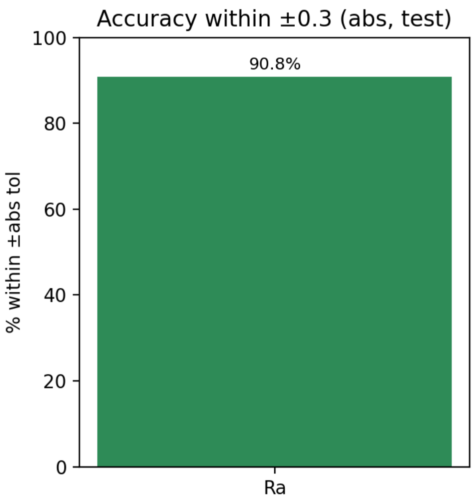

Figure S242: accuracy within tol abs 0p3 ()

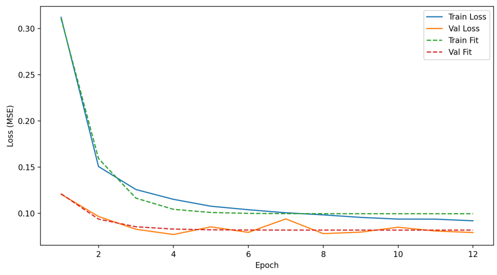

Figure S243: loss curves ()

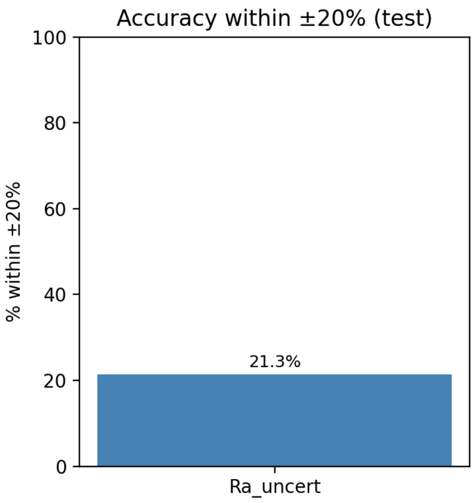

Figure S244: accuracy within tol 20percent ()

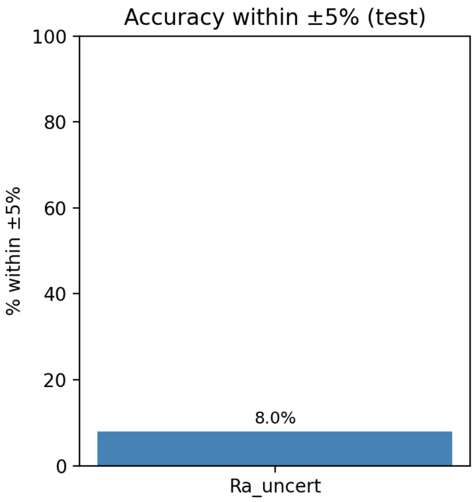

Figure S245: accuracy within tol 5percent ()

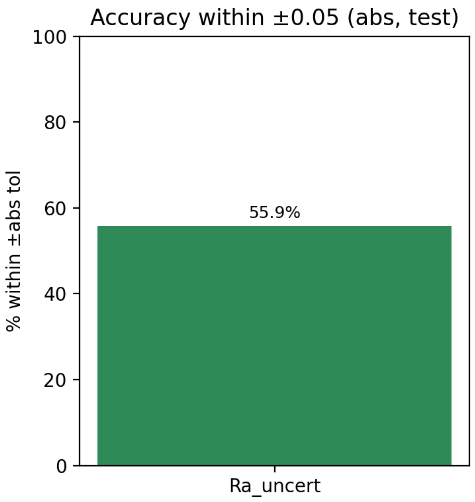

Figure S246: accuracy within tol abs 0p05 ()

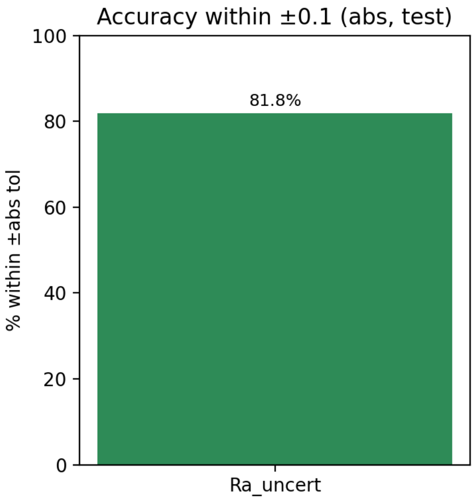

Figure S247: accuracy within tol abs 0p1 ()

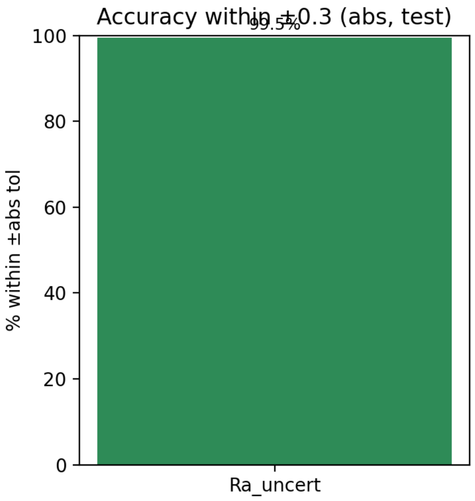

Figure S248: accuracy within tol abs 0p3 ()

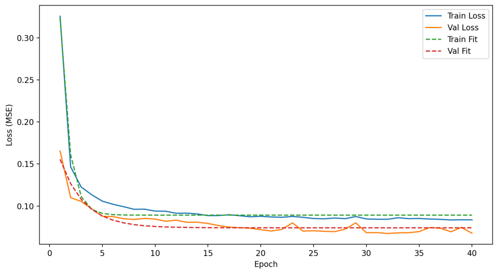

Figure S249: loss curves ()

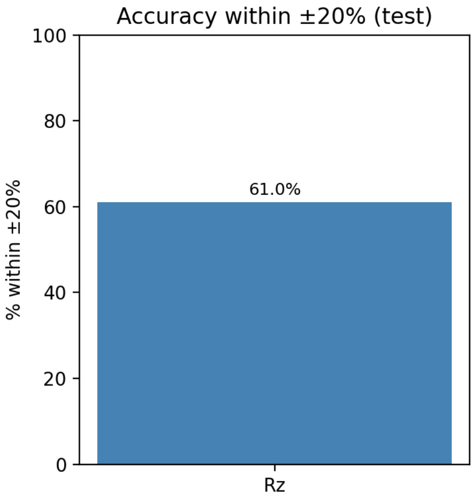

Figure S250: accuracy within tol 20percent ()

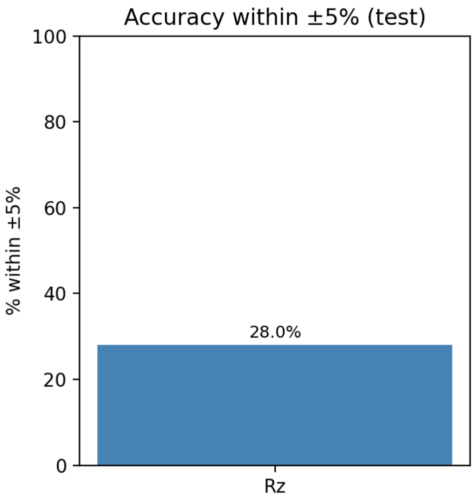

Figure S251: accuracy within tol 5percent ()

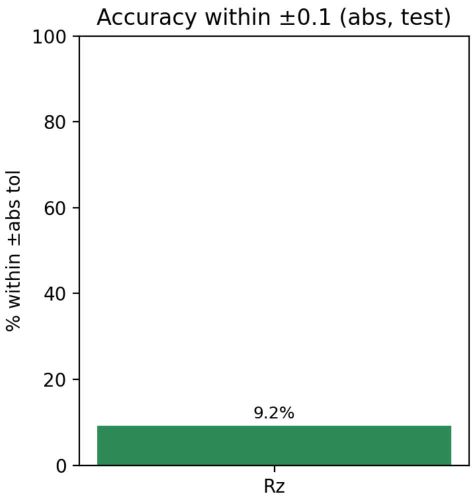

Figure S252: accuracy within tol abs 0p1 ()

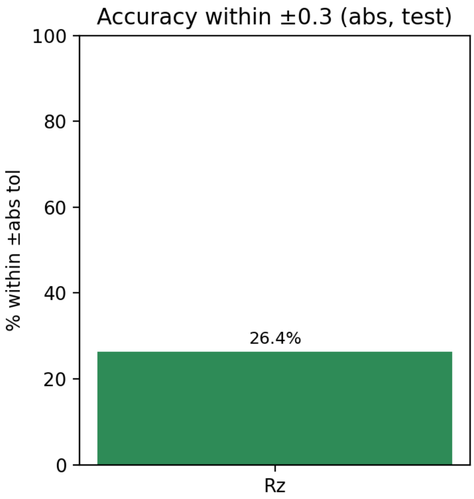

Figure S253: accuracy within tol abs 0p3 ()

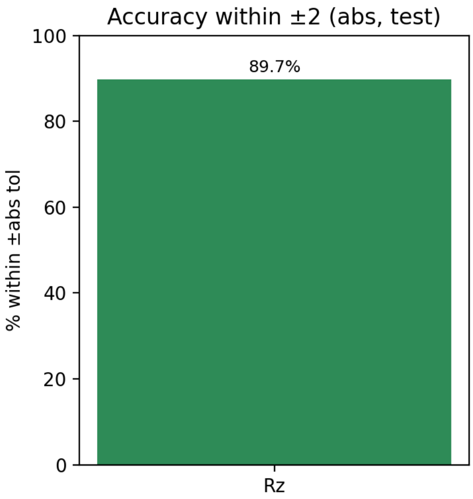

Figure S254: accuracy within tol abs 2 ()

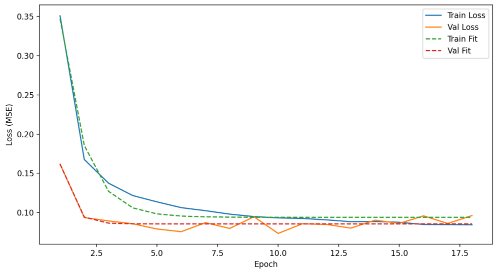

Figure S255: loss curves ()

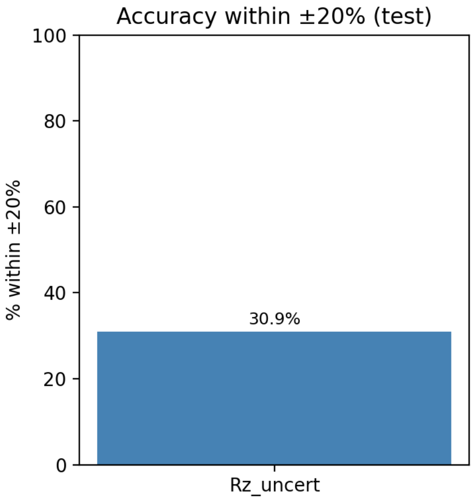

Figure S256: accuracy within tol 20percent ()

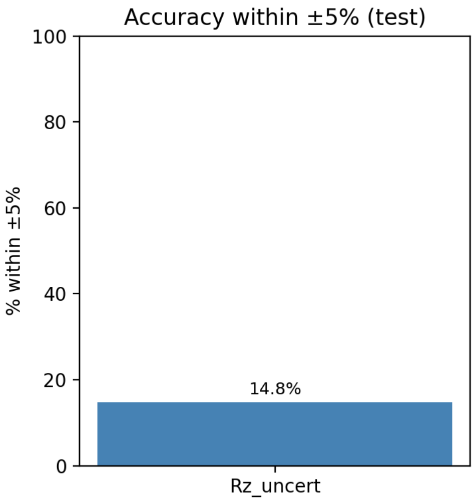

Figure S257: accuracy within tol 5percent ()

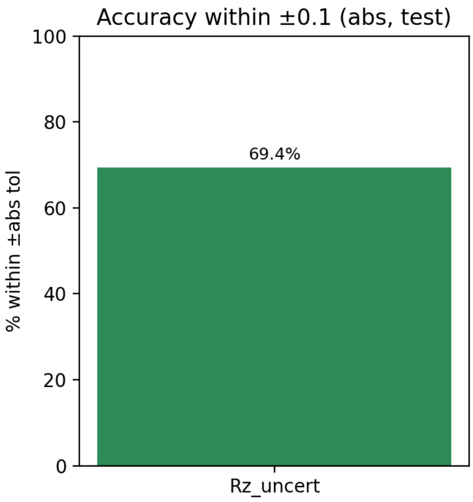

Figure S258: accuracy within tol abs 0p1 ()

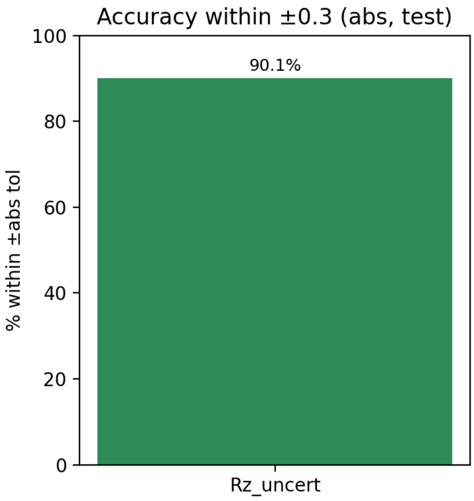

Figure S259: accuracy within tol abs 0p3 ()

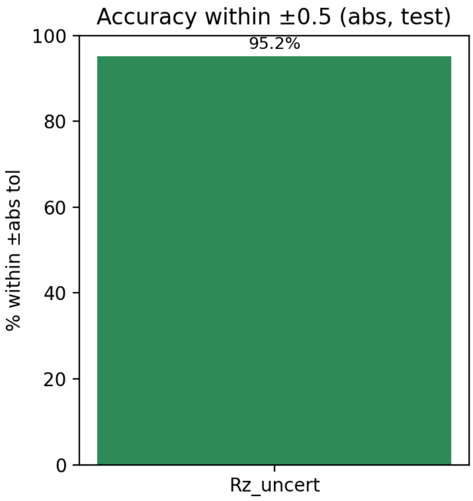

Figure S260: accuracy within tol abs 0p5 ()

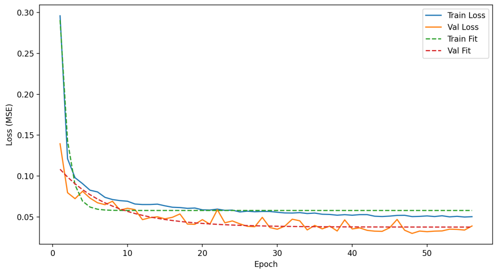

Figure S261: loss curves ()

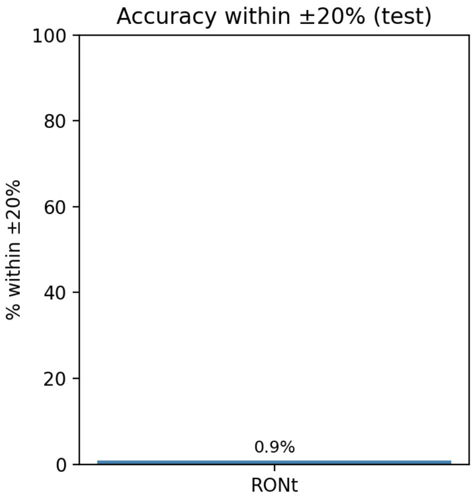

Figure S262: accuracy within tol 20percent ()

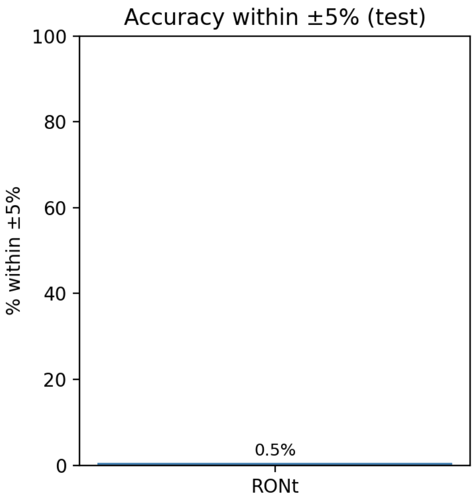

Figure S263: accuracy within tol 5percent ()

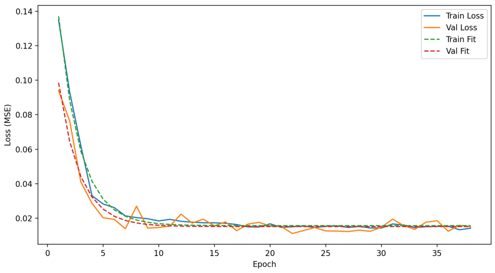

Figure S264: loss curves ()

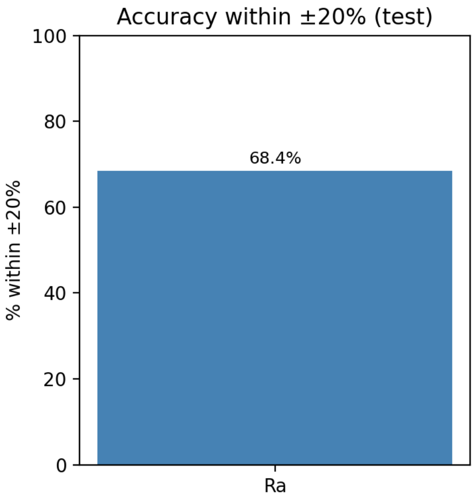

Figure S265: accuracy within tol 20percent ()

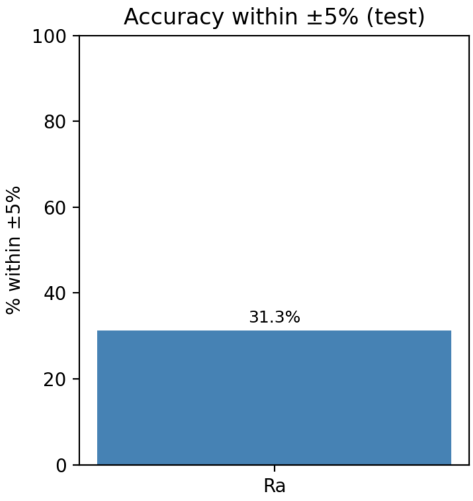

Figure S266: accuracy within tol 5percent ()

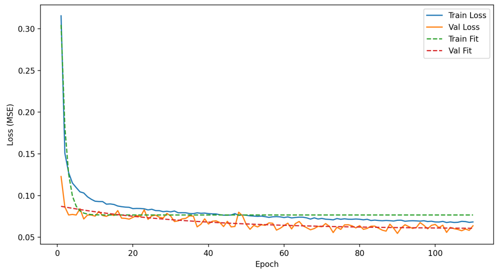

Figure S267: loss curves ()

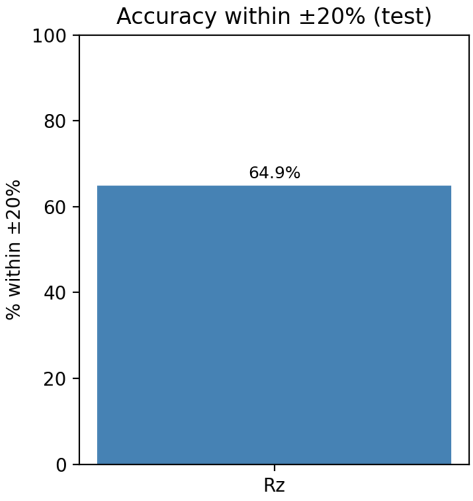

Figure S268: accuracy within tol 20percent ()

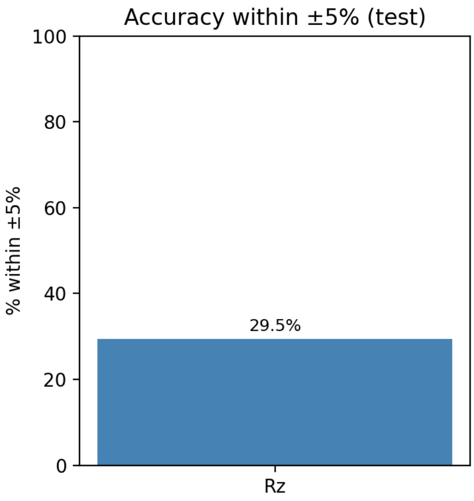

Figure S269: accuracy within tol 5percent ()

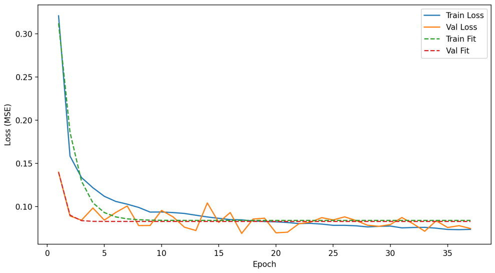

Figure S270: loss curves ()

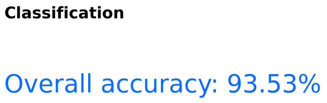

Figure S271: headline classification ()

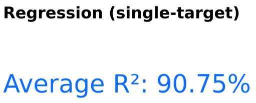

Figure S272: headline regression ()

Supplementary Uncertainty Analysis

This section expands the manuscript's uncertainty discussion with full numeric artefacts derived from the tabular outputs.

A. Interval Coverage and Widths

Quantile empirical coverages and mean widths for central intervals (0.50, 0.80, 0.90 nominal) are summarised in Table S1. Conformal recalibration (Table S2) adjusts widths while restoring nominal coverage.

Table S1. Quantile-derived empirical coverage (EC) and mean width (MW) by target (pre-calibration).

oprule Target	Interval	Nominal (NC)	Empirical (EC)	Mean Width [μm]	Notes
Ra	0.05–0.95	0.90	0.9832	1.2120	Over-coverage
Ra	0.10–0.90	0.80	0.8849	0.7700	Mild over-coverage
Ra	0.25–0.75	0.50	0.5832	0.4018	Over-coverage
Rz	0.05–0.95	0.90	0.3022	3.6751	Severe under-coverage
Rz	0.10–0.90	0.80	0.1890	2.3628	Under-coverage
Rz	0.25–0.75	0.50	0.1003	1.2728	Under-coverage
RONt	0.05–0.95	0.90	0.1507	0.04713	Severe under-coverage
RONt	0.10–0.90	0.80	0.1034	0.03048	Under-coverage
RONt	0.25–0.75	0.50	0.0602	0.01643	Under-coverage
Ra_uncert	0.05–0.95	0.90	0.3092	0.3535	Under-coverage
Ra_uncert	0.10–0.90	0.80	0.1825	0.2237	Under-coverage
Ra_uncert	0.25–0.75	0.50	0.0993	0.1166	Under-coverage
Rz_uncert	0.05–0.95	0.90	0.6554	1.7578	Under-coverage
Rz_uncert	0.10–0.90	0.80	0.5362	1.1324	Under-coverage
Rz_uncert	0.25–0.75	0.50	0.3725	0.6087	Under-coverage
RONt_uncert	0.05–0.95	0.90	0.1496	0.00473	Severe under-coverage
RONt_uncert	0.10–0.90	0.80	0.1029	0.00306	Under-coverage
RONt_uncert	0.25–0.75	0.50	0.0602	0.00165	Under-coverage

EC and NC are shown as fractions (0–1). Widths are reported in [μm]. Empirical coverage exceeding nominal indicates over-coverage; below nominal indicates under-coverage.

Table S2. Conformal 90% central interval coverage (COV) and mean width (MW) vs baseline 90% width; width change Δ expressed as percent.

Target	Quantile Width [μm]	Conformal Width [μm]	Width Δ [%]	Conformal Coverage
Ra	1.2120	0.6701	-44.7	0.9055
Rz	3.6751	3.0517	-17.0	0.9010
RONt	0.04713	2.7e-06	-99.99	0.8987

Conformal coverage is shown as a fraction (0–1). Negative width Δ denotes interval narrowing post conformal recalibration while maintaining nominal coverage.

B. Interval Scoring Metrics

Pinball, CRPS approximation and Winkler scores for the quantile model are detailed in Table S3. Lower is better across metrics.

Table S3. Expanded interval scoring metrics (quantile model).

Target	Mean Pinball	CRPS (approx)	Winkler 80%	Winkler 90%	Note
Ra	0.0561	0.1273	0.8701	1.2404	High coverage 90%
Rz	2.4961	4.7712	48.7100	87.2951	Large scale
RONt	0.0263	0.0514	0.5111	0.8584	Narrow scale
Ra_uncert	0.2361	0.4536	4.6224	8.1124	Learned uncertainty
Rz_uncert	0.3978	0.8188	7.4755	11.2897	Wider dispersion
RONt_uncert	0.00262	0.00513	0.05094	0.08543	Very small variance

All scores are in units of the response variable [μm]. Lower values indicate better probabilistic calibration and sharpness; Winkler penalises mis-coverage and width jointly.

C. Residual Tail Heaviness

Excess kurtosis (Table S4) highlights heavy-tailed error structure, especially for *RONt* and *RONt_uncert*. These motivate future adoption of robust likelihoods or quantile-local conformal adjustments.

Table S4. Excess kurtosis of residuals (test set) for primary targets and direct uncertainty targets.

Target	Excess Kurtosis	Comment
Ra	33.89	Heavy tail vs Gaussian (0)
Rz	34.98	Heavy tail
RONt	176.14	Extreme tail weight
Ra_uncert	2.58	Mild tail elevation
Rz_uncert	39.83	Heavy tail
RONt_uncert	274.71	Extreme tail / degeneracy

Gaussian reference excess kurtosis is 0; large positive values indicate heavy-tailed error distributions.

D. Residual–Uncertainty Correlations

Correlation between absolute residuals and predicted uncertainty targets ($|e|$ vs corresponding predicted uncertainty variable, e.g. `Ra_uncert`) for aligned target pairs is shown in Table S5. A stronger positive value indicates better heteroscedastic signal capture.

Table S5. Absolute residual vs predicted uncertainty correlation coefficients.

Pair	r
|e(Ra)| vs Ra_uncert	-0.054
|e(Rz)| vs Rz_uncert	0.031
|e(RONt)| vs RONt_uncert	0.789

Positive correlation suggests predicted uncertainty scales with realised absolute errors (heteroscedastic signal capture).

Acknowledgements

A grant supported this work: project entitled: "Application of artificial intelligence in surface irregularities measurements", financed by the Ministry of Education and Science of

the programme: Polish Metrology II PM-II/SP/0104/2024/02 of 01.02.2024
Projekt pt. „Zastosowanie sztucznej inteligencji w pomiarach nierówności powierzchni" finansowany przez Ministerstwo Nauki i Szkolnictwa Wyższego w ramach programu Polska Metrologia 2 Nr PM-II/SP/0104/2024/02 z dnia 01.02.2024.

Disclaimer/Publisher's Note: The statements, opinions and data contained in all publications are solely those of the individual author(s) and contributor(s) and not of MDPI and/or the editor(s). MDPI and/or the editor(s) disclaim responsibility for any injury to people or property resulting from any ideas, methods, instructions or products referred to in the content.
